# Single Atom Sites in Ga‐Ni Supported Catalytically Active Liquid Metal Solutions (SCALMS) for Selective Ethylene Oligomerization

**DOI:** 10.1002/cphc.202400651

**Published:** 2025-03-14

**Authors:** Alexander Søgaard, Tzung‐En Hsieh, Julien Steffen, Simon Carl, Mingjian Wu, Yousuf R. Ramzi, Sven Maisel, Johannes Will, Anna Efimenko, Mihaela Gorgoi, Regan G. Wilks, Johannes Frisch, Nicola Taccardi, Marco Haumann, Erdmann Spiecker, Andreas Görling, Marcus Bär, Peter Wasserscheid

**Affiliations:** ^1^ Lehrstuhl für Chemische Reaktionstechnik (CRT) Friedrich-Alexander-Universität Erlangen-Nürnberg (FAU) Egerlandstr. 3 91058 Erlangen Germany; ^2^ CHEC Research Centre, Department of Chemical and Biochemical Engineering Technical University of Denmark (DTU) Søltofts Plads 229 2800 Kongens Lyngby Denmark; ^3^ Department of Interface Design Helmholtz-Zentrum Berlin für Materialien und Energie GmbH (HZB) Albert-Einstein-Str. 15 12489 Berlin Germany; ^4^ Lehrstuhl für Theoretische Chemie Friedrich-Alexander-Universität Erlangen-Nürnberg (FAU) IZNF Egerlandstr. 3 91058 Erlangen Germany; ^5^ Institute of Micro- and Nanostructure Research & Center for Nanoanalysis and Electron Microscopy (CENEM) Friedrich-Alexander-Universität Erlangen-Nürnberg (FAU) Cauerstr. 3 91058 Erlangen Germany; ^6^ Research Centre for Synthesis and Catalysis, Department of Chemistry University of Johannesburg P.O. Box 524 Auckland Park 2006 South Africa; ^7^ Department X-ray spectroscopy at interfaces of thin films Helmholtz-Institute Erlangen-Nürnberg for Renewable Energy (HI ERN) Albert-Einstein-Str. 15 12489 Berlin Germany; ^8^ Department of Chemistry and Pharmacy Friedrich-Alexander-Universität Erlangen-Nürnberg (FAU) Egerlandstr. 3 91058 Erlangen Germany; ^9^ Forschungszentrum Jülich GmbH Helmholtz-Institute Erlangen-Nürnberg for Renewable Energies (IEK 11) Cauerstraße 1 91058 Erlangen Germany; ^10^ Institute for a Sustainable Hydrogen Economy Marie-Curie-Straße 5 52428 Jülich Germany

**Keywords:** Supported catalysts, Single atom sites, Photoelectron spectroscopy, Electron microscopy, Computational chemistry

## Abstract

Supported catalytically active liquid metal solutions (SCALMS) are materials composed of a liquid metal alloy deposited on a porous support. Due to the dynamic properties of the liquid metal alloy, these systems are suggested to form single atom sites, resulting in unique catalytic properties. Ga−Ni SCALMS were successfully applied to ethylene oligomerization, yielding catalysts that were stable up to 120 h time on stream. A workflow based on synchrotron‐based X‐ray photoelectron spectroscopy (XPS), transmission electron microscopy (TEM) as well as density function theory (DFT) and ab initio molecular dynamics (AIMD) simulations was applied to investigate the nature of the active species in these materials. The combination of XPS with DFT calculations indeed indicates the presence of isolated single Ni atoms on the liquid metal surface, while TEM measurements show high dynamics in the liquid metal with intermetallic phase dissolution and transformation. Furthermore, DFT/AIMD methods allowed for rationalizing the role of hydrogen pretreatment in enriching the Ni atom at the surface of the liquid metal alloy.

## Graphical abstract

Supported catalytically active liquid metal solutions (SCALMS) based on liquid gallium‐nickel (Ga−Ni) alloys have demonstrated high catalytic activity and stability in the selective oligomerization of ethylene. In this study of novel Ga−Ni SCALMS, catalytic, spectroscopic, microscopic, and computational methods provide a unique and first insight into the highly dynamic nature of the catalytically active single Ni atom sites in the Ga matrix.

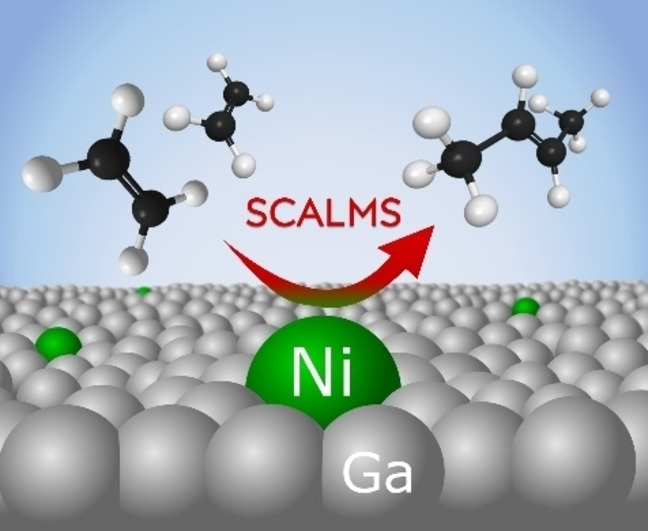

## Introduction

Reactivity on liquid metal surfaces has recently gained a lot of interest for application in electro‐ and thermocatalysis.[^1^, ^2^, ^3^, ^4^, ^5^, ^6^] Supported catalytically active liquid metal solutions (SCALMS) are a new class of catalysts that are composed of liquid alloy droplets supported by a porous oxide support, e. g., SiO_2_ and Al_2_O_3_.^[7]^ The alloys consist of a low‐melting‐point metal as liquid metal matrix, e. g., Ga, In or Sn in which a small amount of a catalytically active metal, e. g., Pd, Pt or Rh is dissolved.[^1^, ^3^, ^8^, ^9^, ^10^] In contrast to conventional homogeneous catalysis with supported liquid phases, the catalytic reaction in SCALMS occurs only at the molten metal/gas interface, as the liquid metal does not provide relevant solubility for the organic reactants. The liquid nature of the supported alloy droplets under the reaction conditions has been confirmed through a combination of X‐ray diffraction (XRD), neutron scattering, X‐ray photoelectron spectroscopy (XPS), and molecular dynamic (MD) calculations.[^1^, ^3^, ^7^, ^11^] Catalytic experiments, spectroscopic methods and DFT calculations have shown that the active metal is depleted at the interface and thus emerges as single atom site at the surface during catalysis.[^12^, ^13^] SCALMS catalysts exhibited very promising performances in alkane and cycloalkane dehydrogenations, as shown in particular for Ga–Pd,^[3]^ Ga–Rh,^[1]^ and Ga–Pt[^7^, ^14^, ^15^, ^16^] systems. Recently, the formation of single atom sites in liquid metals has been also suggested for Ga−Pt and Ga−Cu systems, for electro‐chemical methanol oxidation and ammonia production, respectively.[^5^, ^6^] In addition, recent study indicates that the composition of liquid matrix, i. e., changing the matrix from Ga to binary GaSn and GaIn, also have impact on catalytic performance of SCALMS, which indicates a promising method for catalysts modification.^[8]^ In fact, the SCALMS concept provides a neoteric approach to single‐atom catalysis, a concept that has recently attracted much interest in the catalysis community.[^17^, ^18^, ^19^]

Here, we present detailed structural studies of Ga−Ni SCALMS systems and their application in the field of ethylene oligomerization. The oligomerization of lighter alkenes is of high industrial interest.[^20^, ^21^, ^22^] While this reaction is well‐known to be promoted by homogeneous catalysts,[^23^, ^24^, ^25^] literature describes only few examples of heterogeneous catalysis, such as e. g., mesoporous Ni‐aluminosilicates and Ni‐exchanged zeolites.[^20^, ^26^, ^27^, ^28^]

Recently, some of us reported Ga−Ni SCALMS as an active and stable catalyst system for ethylene oligomerization.[^29^, ^30^] The reported Ga−Ni SCALMS system was characterized by high dimerization activity, selectivity, and remarkable stability during operation. The catalytic activity was found to be tightly linked to the temperature range where a liquid state of the Ga−Ni alloy was expected from the phase diagram. Under these conditions, Ga−Ni SCALMS systems were found to outperform both partially liquid Ga−Ni alloys and solid intermetallic phases as well as pure Ni nanoparticle‐based catalysts. Based on the analogy with other SCALMS systems, it was hypothesized that the isolated Ni atom sites in a Ga matrix would be responsible for the remarkable catalytic activity of the Ga−Ni SCALMS system.^[29]^

In this contribution, we present unprecedented insight into the electronic and dynamic properties of this Ga−Ni alloy system to further elucidate its catalytic behavior.

## Results and Discussion

### Catalysis Studies of Ga−Ni SCALMS in Ethylene Oligomerization

**Figure 1:**
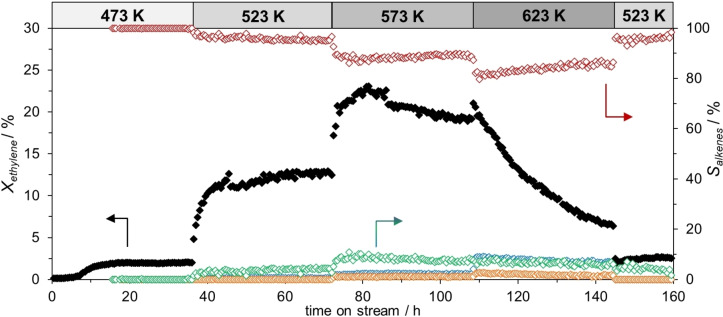
Ethylene oligomerization using Ga_67_Ni/SiO_2_ at temperatures ranging from 473 to 623 K. Left axis: conversion (*X*_ethylene_) (black closed), right axis: selectivity (*S*_alkenes_) for C3 (blue open), C4 (red open), C5 (orange open) and C6 (green open). Reaction conditions: 473 K (0–36 h TOS), 523 K (37–72 h TOS), 573 K (73–108 h TOS), 623 K (109–144 h TOS), 523 K (145–160 h TOS), 0.6 MPa, 1.0 g_catalyst_, 3000 mL_N_ g_catalyst_^−1^ h^−1^, 20 vol% C_2_H_4_ in He, no pretreatment.

We start by demonstrating the effect of different reaction temperatures on the catalytic performance of a 1.5 at % Ni in Ga SCALMS on a SiO_2_ support (Ga‐to‐Ni atomic ratio of 67, Ga_67_Ni/SiO_2_) in continuous ethylene oligomerization over a total time‐on‐stream (TOS) of 160 h (Figure 1). In this experiment, the unpretreated material was exposed to 0.6 MPa of diluted ethylene (20 vol% C_2_H_4_ in He) and the temperature was increased stepwise from 473 K to 623 K. At 473 K, the material was barely active, with a ca. 2 % conversion reached after an induction time of almost 10 h. In accordance with our previous investigation,^[29]^ Ga−Ni SCALMS exhibited significant catalytic activity in the oligomerization of ethylene starting from 523 K, showing first a steep activation phase, followed by a flatter activation profile.

Expectedly, the activity increased further when operating at 573 K, reaching a maximum conversion of 23 %. However, under these conditions, the catalyst was found to deactivate, and the deactivation was even much more pronounced at the highest applied temperature of 623 K. Subsequent lowering of the temperature to 523 K did not restore the previous catalytic performance, indicating an irreversible deactivation. At the same time, propene (C3) and pentenes (C5) were detected in the product stream at temperatures above 573 K. Likewise, small amounts of coke were detected after reaction at 623 K (see SI, Figure S1), suggesting that the catalyst exhibited some cracking activity at temperatures above 573 K.

**Figure 2:**
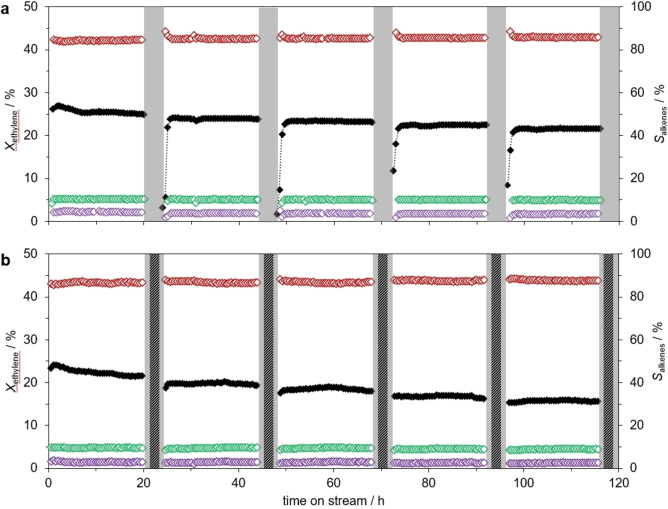
Catalytic performance of Ga−Ni SCALMS in ethylene oligomerization through periodic purging of (a) He over Ga_75_Ni/SiO_2_ and (b) H_2_/He over Ga_49_Ni/SiO_2_. Left axes: conversion (*X*_ethylene_) (black closed), right axes: selectivity (*S*_alkenes_) for C4 (red open) and C6 (green open) and C8 (purple open). C3 was <1 % and is not shown. Reaction conditions: Ethylene oligomerization (white area): 20 h TOS, 533 K, 0.6 MPa, 1.0 g_catalyst_, 3000 mL_N_ g_catalyst_^−1^ h^−1^ (GHSV=490 h^−1^), 20 vol% C_2_H_4_ in He. Periodic He purging (light grey area): 553 K, 0.6 MPa, 12 L_N_ g_catalyst_^−1^ h^−1^, 100 vol% He, 4 h TOS in (a) and 2 x 1 h TOS in (b). H_2_/He purging (dark grey area): 553 K, 0.6 MPa, 6 L_N_ g_catalyst_^−1^ h^−1^, 20 vol% H_2_ in He, 2 h TOS in b). Pretreatment conditions (for both (a) and (b)): 2 h TOS, 583 K, 0.1 MPa, 1200 mL_N_ g_catalyst_ h^−1^, 100 vol% H_2_.

Our previous studies on support effects and coke formation in SCALMS systems[^7^, ^14^, ^31^] showed that on SCALMS the formation of coke at the liquid metal interface is significantly reduced. This property is ascribed to the high dynamics of the liquid alloy droplets as well as the lack of vicinal active sites at the interface, which are essential for coke formation.[^1^, ^10^] Thus, the minor amount of coke observed on SCALMS catalysts should be predominantly formed on the support surface.^[7]^ Alternatively, the observed deactivation might be induced by accumulation of high‐boiling oligomers in the catalyst material.[^20^, ^32^, ^33^] To shed light on the possible deactivation mechanism, the byproduct condensation was evaluated by periodic purging with an inert gas (i. e. He) to flush out high‐boiling olefin products. Figure 2a shows the catalytic performance of a Ga−Ni SCALMS on silica with a Ga : Ni‐ratio of 75 (Ga_75_Ni/SiO_2_) during five cycles of 24 h each.

The material was tested using optimized temperature conditions: It was first pretreated with H_2_ at 583 K and then operated at 533 K. Hydrogen pretreatment was shown to eliminate the initial induction period and to boost the catalytic activity.[^29^, ^30^] Most remarkably, the system exhibited an initial conversion above 25 % and showed steady‐state performance during each cycle, corresponding to a Ni‐based productivity of above 240 g_oligomers_ g_Ni_^−1^ h^−1^. All catalysts presented in this work exhibited similarly high productivities when operated under these optimized conditions. Figure 2b shows the result with a Ga_49_Ni/SiO_2_ system reaching 280 g_oligomers_ g_Ni_^−1^ h^−1^ initial productivity at 533 K and 0.6 MPa (see further details in SI, Table S1). After 5 cycles (120 h TOS in total), the conversion level reached 22 %, with slight deactivation occurring after each He purging. The selectivity for C4, C6 and C8 remained constant throughout the experiment at 86 %, 10 % and 4 %, respectively, which regarding selectivity is in good accordance with our previous study.^[29]^

However, as no full recovery of the catalytic activity after He purging was observed, it is unlikely that condensation of higher oligomers is the main cause of the observed catalyst deactivation. Interestingly, the activity of the system was found to be significantly lower immediately after the He purging, however, resuming a higher activity level after a short induction phase (Figure 2a). Notably, when H_2_ was supplied during the purging step (Figure 2b), such induction phases were not observed. This indicates that He purging changes the reactive interface to a less active but reactivatable state, whereas H_2_ is able to retain the catalytically active Ni species at the reactive interface. Moreover, it is evident that H_2_ plays a significant role in the retention of active species at the liquid metal/gas interface. However, it was also observed that the decrease in activity during the purging cycles was more pronounced in the presence of H_2_, compared to the purely inert He atmosphere.

To explain these observations and to shed light on the nature of the active site in Ga−Ni SCALMS, we report in the following results of analytical studies that were carried out using relevant Ga−Ni model samples prepared via physical vapor deposition on a silicon wafer with a native SiO_2_ layer (PVD; See SI for details).

### Effects of Ni Isolation on the Electronic Structure of the Ga−Ni Alloy Surface

**Figure 3:**
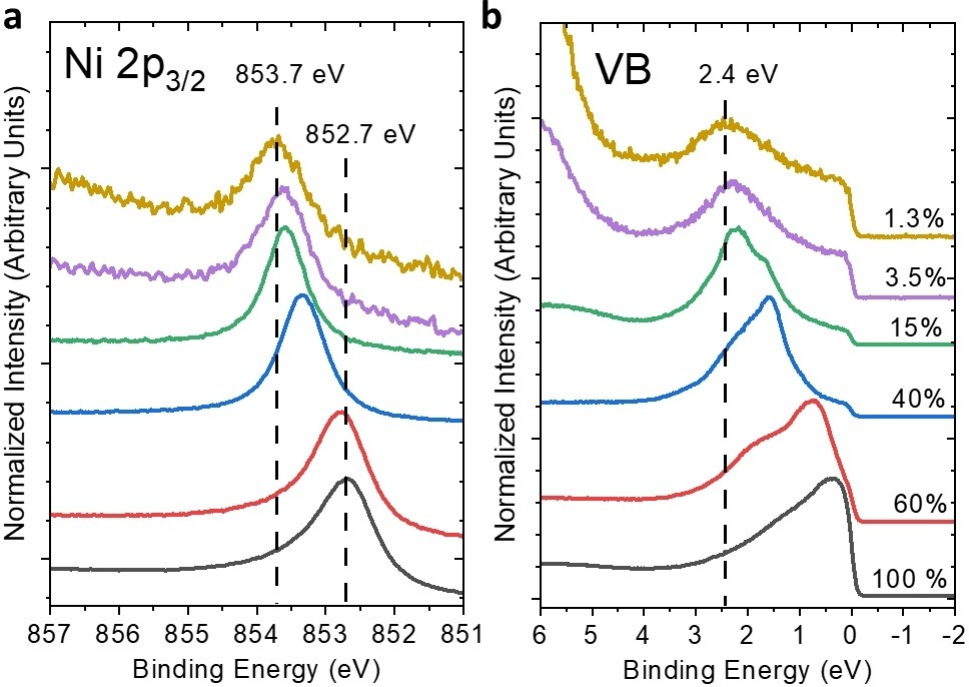
Synchrotron‐based XPS spectra of Ni 2p_3/2_ (a) and valence band (VB) spectra (b) of Ga−Ni alloys with different Ni concentrations in at % measured at room temperature. The difference between nominal and calculated Ni concentration is shown in Figure S2–S4 and Table S2‐S3 in SI. The photon energy of the incident X‐rays is set to 953 eV and 100 eV for Ni 2p and VB measurements, respectively, resulting in a similar kinetic energy (of ≈100 eV) of the probed photoelectrons ensuring the same information depth for the Ni 2p and VB XPS measurements.

A series of Ga−Ni alloys with different contents of Ni by synchrotron‐based X‐ray photoelectron spectroscopy (XPS) were investigated to address the effect of site isolation on the chemical and electronic structure of the Ni atoms in the Ga matrix (Figure 3).

The observed trend of peak shift is counterintuitive, as Ni atoms are expected to get negatively charged by charge transfer from Ga. Hence one would expect a peak shift to lower BE (cf. Bader charge calculation in Figure 6b). The observed trend can be rationalized as a diminished screening effect on generated core holes within the Ni atoms, which overcompensates the influence of negative charge on Ni atoms as nicely reproduced by the DFT calculations (FS‐IS shift) in Figure 6b. The same phenomenon was observed in Ga−Rh alloys showing that the screening of the created Rh‐related core holes is less efficient for isolated Rh.[^9^, ^34^] Moreover, we observe a narrowing and shift to higher BE of the Ni 3 d derived states in the valence band as a function of decreasing Ni concentration in the Ga−Ni alloys (Figure 3b).

According to previous studies in Ga−Rh and Ga−Pd systems and the DFT calculations discussed below, such variation of the d‐band feature can be regarded as an indication of the presence of isolated transition metal sites.[^9^, ^35^, ^36^] Based on the calculated inelastic mean free path (IMFP) of photoelectrons with a kinetic energy of 100 eV (0.5 nm)[^37^, ^38^, ^39^], we propose that the observed narrow d‐band of the 1.3 at % Ni sample is associated with isolated Ni atoms emerging at the surface of Ga−Ni alloys.

**Figure 4:**
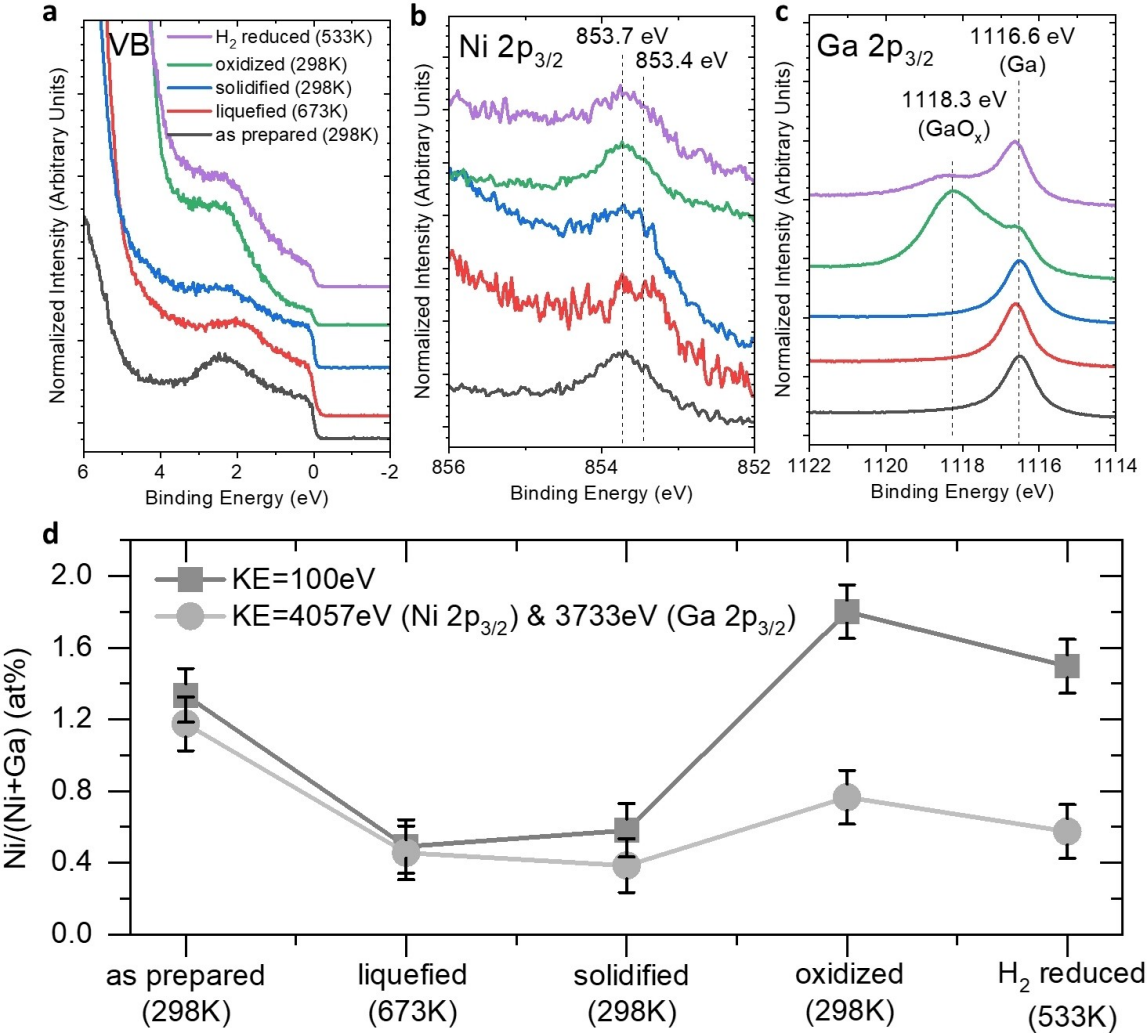
Synchrotron‐based XPS spectra (KE=100 eV) of VB (a), Ni 2p_3/2_ (b), and Ga 2p_3/2_ spectra (c) of a Ga−Ni alloy with 1.3 at % Ni measured at different temperatures/after different sequential post‐deposition treatments. Panel (d) represents the evolution of the XPS‐derived Ni/(Ni+Ga) ratio in the Ga−Ni sample for different temperatures/at different post‐deposition treatment steps measured for 2 different probing depth (that correspond to IMFP values of 0.5 and 5.0–5.3 nm) of the detected Ga 2p_3/2_ and Ni 2p_3/2_ photoelectrons.

After examining the spectroscopic fingerprints of isolated Ni atoms, we gained insights into how the isolated Ni atoms behave under reaction conditions. Ga−Ni SCALMS catalysts are generally exposed to ambient conditions during catalyst synthesis and storage,[^7^, ^29^] pretreated with H_2_, and operated at elevated temperatures (i. e., 533 K). Hence, the influence of liquefaction, oxidation, and reduction of the Ga−Ni surface was investigated. Corresponding sequential post‐deposition treatments of a 1.3 at % Ga−Ni sample were conducted to study the evolution of the electronic structure of the Ni atoms. All spectra did not show any significant contamination during these post‐treatments (see SI, Figure S6). We followed the spectral fingerprint of isolated Ni atoms in the Ni 2p_3/2_ and VB spectra after each treatment (Figure 4). First, the sample was measured at 673 K to study the chemical and electronic structure of the sample in a fully liquefied state. 22 The liquefaction caused the Ni 3d‐derived region in the VB spectrum to broaden and its center to shift 0.3 eV to lower BE (Figure 4a). Note that the liquefaction was also accompanied by the expected dewetting of the deposited Ga−Ni alloys which is indicated by the increased intensity of the substrate‐related Si 2p core level (see SI, Figure S7). Similar BE shift and broadening were observed in the Ni 2p_3/2_ spectrum. Fitting the XPS‐/ hard X‐ray photoelectron spectroscopy (HAXPES) data (see SI, Figure S11 and S12) reveals the presence of new Ga−Ni phases at 673 K.

According to the TEM investigation (vide infra), these intermetallic phases also contain isolated Ni atoms surrounded by Ga atoms, e. g., Ga_7_Ni_3_. Furthermore, the corresponding spectral features of the Ni 2p_3/2_ line are more pronounced in the bulk‐sensitive data, indicating that the new Ga−Ni phases are located mainly in the bulk (see SI, Figure S11 and S12).

Noteworthy, the Ni 2p_3/2_ feature shifts to the previous position after cooling down to 298 K (Figure 4b). Such reversible formation and dissolution of Ga−Ni phases were also observed by TEM analysis (*vide infra*). After solidification, the sample was exposed to 10^−3^ Pa O_2_ flow for 30 min, causing the formation of a GaO_x_ shell (Figure 4c), with no significant effect on the Ni 2p_3/2_ spectra (i. e., no spectral evidence of Ni oxide formation). Subsequently, the sample was reduced in 10^−3^ Pa of H_2_ at 573 K for 30 min, and then measured at 533 K, in order to mimic the reaction conditions applied for real SCALMS catalysts. This decreased the GaO_x_ signal intensity by 70±5 % (Figure 4c and Figure S9, Figure S10 and Table S4 in SI). The spectroscopic fingerprint of isolated Ni atoms (i. e. main Ni 2p_3/2_ contribution at 853.7 eV) is always observed in the XPS data during these experiments (see fits in SI, Figure S11 and S12), indicating the steady presence of isolated Ni atoms in the outer shell of Ga−Ni alloys (Figure 4a and b). The surface Ni/(Ni+Ga) ratio varies upon performing these post‐deposition treatments (Figure 4d). While liquefaction caused a relative decrease of Ni concentration (by approx. 50 %) both at the surface probed by XPS and the sublayers region probed by HAXPES (corresponding spectra Figure S8) up to 15 nm below the surface,[^37^, ^38^, ^39^] the oxidation results in an enrichment of Ni atoms with respect to Ga matrix and this does not change significantly upon the subsequent H_2_ reduction (Figure 4d, and Table S5 in SI). Note that the calculated Ni/(Ni+Ga) from spectra with KE=500 eV (IMFP=1.2 nm, Figure S13) shows a similar trend as the results of KE=100 eV, shown in Figure 4d. The decrease of Ni content upon liquefaction can be explained by formation of Ni‐rich Ga−Ni intermetallic phases, e. g., Ga_7_Ni_3_ as observed by TEM (*vide infra*). The fact that the Ni contents derived by XPS and HAXPES agree, indicates that the intermetallic phases are located deep within the Ga−Ni alloy, i. e., beyond XPS and HAXPES probing depth resulting in a low Ni/(Ni+Ga) ratio calculated by HAXPES data. The observation that there is no significant Ni depletion upon H_2_ reduction agrees with the MD simulations, showing that the presence of H_2_ fix Ni atoms at the alloy surface (*vide infra*).

### Structural Investigations of Ga−Ni SCALMS Alloys

We further studied the phase behavior, morphology and structure of a model Ga−Ni sample utilizing correlative *in situ* TEM imaging and diffraction. A sample with nominal 2 at % Ni was prepared *via* PVD (see online methods) on MEMS‐based heating chips with SiN_x_ membrane windows (DENSsolution wildfire system)[^40^, ^41^] suitable for *in situ* TEM investigations.

**Figure 5:**
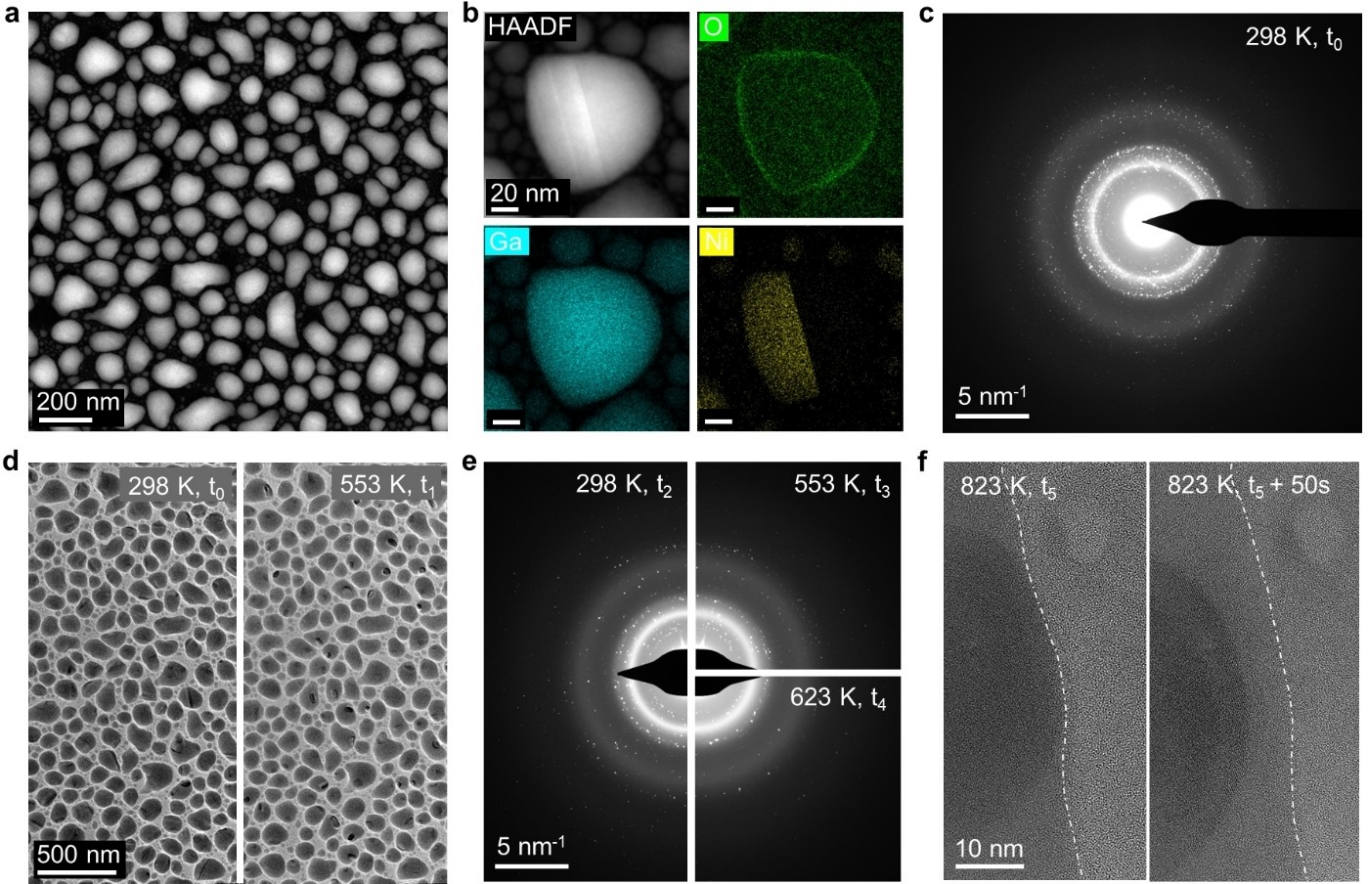
Structure, composition, and phase properties of a nominal Ga_49_Ni sample on a SiN_x_ membrane (a‐c) as‐deposited and (d‐f) at elevated temperatures studied in the TEM. a) HAADF‐STEM Z‐contrast image. b) Elemental EDX maps of O, Ga and Ni (net signals) evaluated based on STEM‐EDX dataset. (c) Selection area electron diffraction (SAED) of the as‐deposited sample. (d) Snapshots of in situ BF TEM imaging at RT and at 553 K. (e) Snapshots of in situ SAED at 298 K, 553 K and 623 K. (f) Snapshots of in situ HRTEM taken at 823 K. Time markers in Figure S16.

As shown in the STEM Z‐contrast image (Figure 5a), the morphology of the particles consists of irregularly shaped droplets of 20 – 200 nm containing brighter solid intermetallic precipitates. This is further confirmed by the elemental maps (Figure 5b) derived from energy dispersive X‐ray spectroscopy (EDX) as well as by the rich diffraction signal in selection area electron diffraction (SAED) (Figure 5c and SI, Fig. S14 and Movie S1). A few nm thick, amorphous gallium oxide shell is observed in HRTEM image (see SI, Figure S15 and **Movie S2**) and via the EDX map of oxygen (Figure 5b). This shell forms during sample transfer from the PVD furnace to the TEM under ambient conditions. Such droplet morphologies with lamella‐like intermetallic precipitates and an oxide shell were also found in our earlier studied systems of Ga−Rh,[^1^, ^34^, ^42^] Ga−Pd,^[43]^ and Ga‐Pt^[44]^ are thus common for as‐deposited Ga‐rich PVD films. In order to monitor the morphological and structural character of the droplets at the operation temperatures of the SCALMS system in ethylene oligomerization catalysis (473 – 623 K), in situ heating in the electron microscope and investigations up to 823 K were performed in both imaging and diffraction mode (following the temperature history shown in SI, Figure S16). Upon stepwise heating to 553 K, the motion of the solid phases started when the temperature was around 323 K (see SI, **Movie S3**), indicating the high dynamics within the droplets. However, the sample geometry and contrast mechanism does not allow single catalyst sites to be resolved. At temperatures above 373 K, the lamella‐like intermetallic precipitates started to shrink and eventually disappeared in some of the droplets while round‐shaped precipitates started to form at temperatures of 523 K. The observed phase transformation persisted until ~553 K (Figure 5d). According to the Ga−Ni phase diagram, this could be attributed to the peritectic transformation of the low‐melting Ga_5_Ni phase to the high‐melting Ga_7_Ni_3_. These results match the spectroscopic observations discussed in conjunction with Figure 4, and Figure S11 and Figure S12 in the SI, which also indicate the formation of Ni‐rich Ga−Ni phases. However, due to the complexity of the possible phases^[45]^ and overlapping diffraction signals (oxides, (liquid) Ga and SiNx), unambiguous determination of the phases from SAED alone was not feasible. When comparing the SAED patterns after the first heating cycle with the initial one (Figure 5c and e, left half), fewer diffraction spots were visible. This suggests that Ni intermetallic phases indeed dissolved into the liquid Ga. These dissolution/precipitation phenomena of intermetallic compounds further support the co‐existence of several Ga−Ni phases that potentially all contribute to the catalytic activity. Furthermore, in situ diffraction clearly revealed that the diffuse diffraction ring at ~4.2 nm^−1^, which is close to the expected peak position of disordered Ga,[^1^, ^46^] was becoming broad and lower, indicating a phase transition of solid amorphous Ga to liquid Ga after the first heating cycle. The observed behavior is similar to the behavior documented for the Ga−Rh system.^[42]^ Motion of the intermetallic phases away from the edge of the particle was also observed (Figure 5f, right half).

Overall, the *in‐situ* TEM investigations revealed that Ga−Ni intermetallic particles are highly dynamic already at temperatures around 323 K. A phase transformation takes place at temperatures ranging from 373 K to 553 K. The locally different chemical compositions within each droplet caused the shifting of the phase equilibria and phase transitions, resulting in the observed temperature range for the phase transformations. The TEM investigations suggest that low‐melting intermetallic phases work as reservoirs, which supply Ni to the liquid Ga at a mildly elevated temperature. Eventually, phase transition to high melting phase may cause irreversible loss of the availability of Ni for the catalysis explaining the observed deactivation at 623 K shown in Figure 1.

### Computational Investigations of the Ga−Ni Alloy System

To shed more light on the atom‐resolved properties of the Ga−Ni system, periodic DFT simulations were performed with the VASP program package and the PBE functional (details in the SI). First, Ga−Ni random alloys were studied: 256 atoms were set up in a cubic Ni fcc fig. bulk cell with gradual replacement of Ni atoms by Ga atoms, with their geometries reoptimized every 10 at % Ni concentration change.

**Figure 6:**
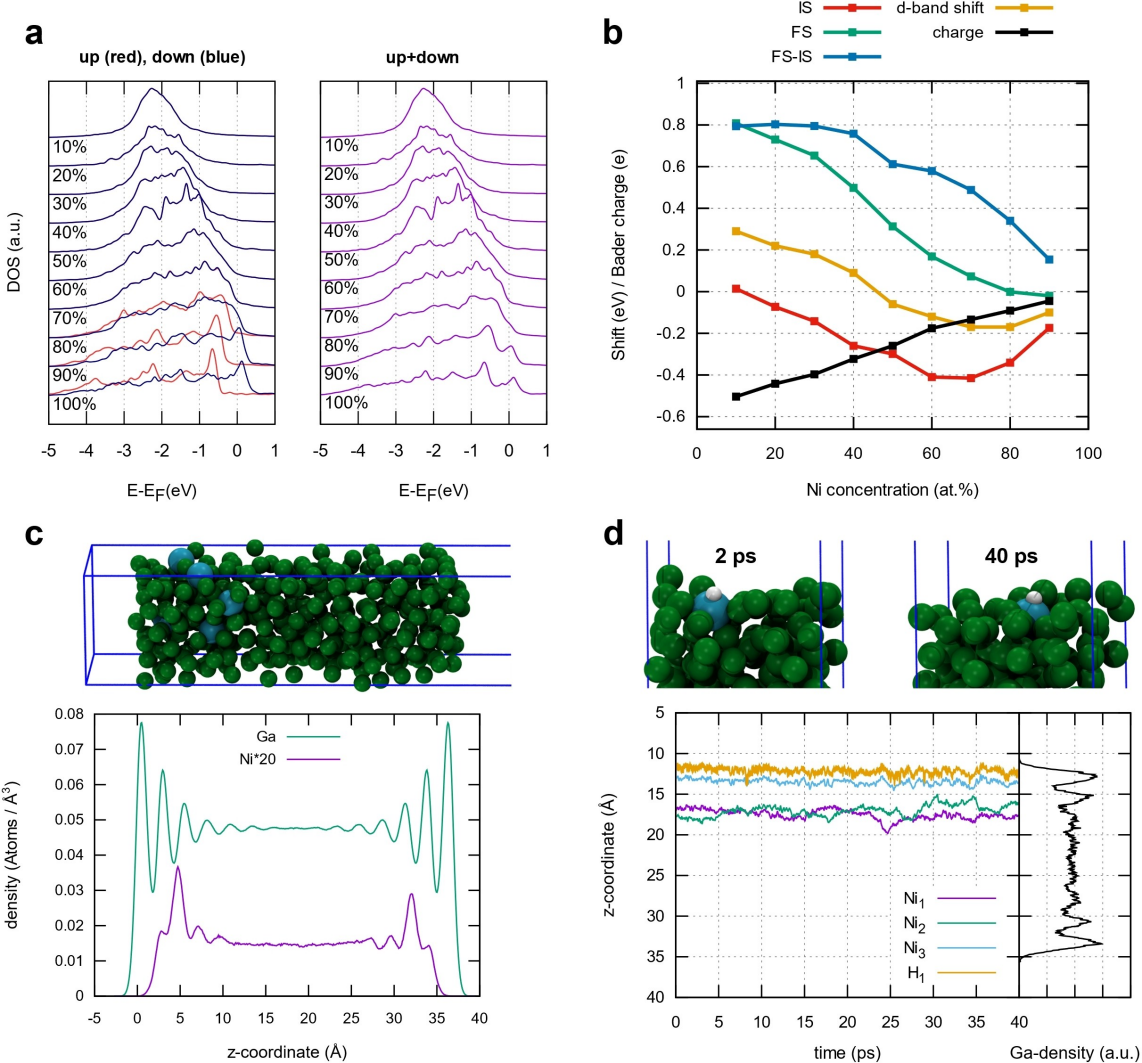
Selected simulation results obtained for the Ga−Ni system. (a) Partial density of states for the Ni 3d band, shown are up and down spin components separately as well as the overall density of states. (b) Initial state (IS) and final state (FS) core level shifts of the Ni 2p level, the difference between the two (FS‐IS) and the d‐band shift, all with respect to a pure Ni reference for different Ni concentration in the random alloys, shown together with the Ni Bader charges. (c) Ga and Ni element densities at 533 K, obtained from ML‐FF sampling trajectories of a surface slab model (a screenshot is shown above the plot, Ga‐atoms: green, Ni‐atoms: blue). (d) Time‐dependent z‐coordinates of H and three Ni atoms (Ni1, Ni2 and Ni3) obtained from an AIMD trajectory of a H adsorbed on Ni3 at the surface of a Ga−Ni slab, together with two screenshots of the surface during the dynamics.

It can be seen in Figure 6a that spin polarization of Ni is significant in the systems containing 80 % or more Ni, for 60 at % or less, on the other hand, the polarization is quenched completely by the surrounding Ga (details concerning magnetic moments and optimized lattice constants are shown in the SI, Table S6). It can thus be expected that SCALMS with even lower Ni concentrations (below 10 at %) show no magnetic behavior. The electronic properties of the random alloys are plotted in Figure 6b. The average charge on Ni is negative for all concentrations, showing a linear increase of a negative charge when going to a lower Ni fraction. This behavior is expected and can be explained by the slightly higher electronegativity of Ni compared to Ga. Due to the negative charge at Ni, the initial state core level shift (IS‐CLS) is always negative, however, not mirroring the linear relation of the charge. In particular, the shift to lower binding energies is increasing from 90 to 70 at %, reaching a minimum between 60 and 70 at % and is then increasing again, reaching a CLS of almost 0 at 10 at %. However, the IS shift nicely mimics the trend of the Ni 3 d band center shift compared to pure Ni. This agrees with previous reports in the literature for Ga‐Rh^[1]^ alloys and Ag‐Pd[^47^, ^48^, ^49^] systems. When final state effects are included (Figure 6b), a shift to higher binding energies can be observed for all samples, being in good agreement with the experimental spectroscopic results (Figure 3). The difference between IS and final state core level shift (FS‐CLS) gives an estimate of the magnitude of the final state effect as compared to pure Ni. This shows that for lower Ni concentration the effect of diminishing core hole screening is enhanced. The modified screening can be attributed to a different band character at the Fermi edge, compared to higher Ni concentrations.

By looking again at the 3d bands in Figure 6a, the shape and width of the d bands change significantly from high to low Ni concentrations, which in turn alters the position of the d band center. This trend matches the spectroscopic observations which also show a shift of Ni 3d band to higher binding energy when Ni is diluted in the Ga matrix. Its width decreases due to the reduced number of Ni nearest neighbors and therefore the increasing degree of site isolation of Ni atoms. This is accompanied by a higher filling of the d band, which arises from the increasing charge transfer of Ga to Ni with rising Ga content. Moreover, the structure of the Ga−Ni surface was modelled. The Ga and Ni element densities orthogonal to the surface were studied with the VASP machine learning force field (ML‐FF), which enabled us to obtain a highly converged density profile for a slab of 335 Ga and 5 Ni atoms after 100 ns of simulation time, far out of reach for ab initio molecular dynamics (AIMD) (Figure 6c). The Ni abundance near the surface is decreased, the upmost layer is built almost completely of Ga atoms. However, in the second and third surface layers, Ni is enriched, with its global density maximum located just 5 Å below the surface. Similar density profiles were already obtained for other binary Ga‐based SCALMS systems such as Ga‐Pd^[3]^ or Ga−Rh.^[1]^ Single Ni atoms nevertheless appear occasionally directly at the surface for some ps (see SI, Figure S17). At higher temperatures, the Ni density near the surface increases (see SI, Figure S18 and Table S7), which is in contrast to the surface Ni depletion revealed in XPS and TEM measurements (Figure 4d). This disagreement can mainly be explained by the experimentally observed Ga−Ni intermetallic phases formation/dissolution. The electronic properties of Ni atoms at the surface are similar to the bulk random alloys for 10 at % Ni; after structural relaxation, however, a slight decrease of core levels near the surface can be seen (Figure 6a, and Figure S19 and Figure S20 in SI).

The influence of hydrogen on the Ga−Ni surface structure was also investigated by AIMD trajectories. In Figure 6d, a Ga−Ni surface slab with three Ni atoms (Ni1, Ni2 and Ni3) is shown. When a single H atom is placed on a Ni near the surface (Ni3), it stays connected to it during the dynamics and prevents Ni3 from disappearing into the bulk, while the two other Ni atoms in the system (Ni1 and Ni2) move to the density maximum 5 Å below the surface. The H−Ni3 complex stays for at least 40 ps at the liquid‐gas interface and thus remains available for catalysis. Other simulations (see SI, Figure S22–S25) have shown that H_2_ molecules placed on Ni mostly desorb directly to the gas phase, but sometimes their bond gets broken by the Ni, and in this case, one H stays on the Ni and the other moves around on the Ga surface. In other words, the Ni atoms are locked on the surface by adjacent H atoms (or an H_2_ molecule) approaching from the gas phase. Finally, this could explain the beneficial effect of hydrogen treatment.

## Conclusions

In summary, our work enables a detailed view on the working principle of Ga−Ni SCALMS in the industrially highly relevant ethylene oligomerization reaction. The catalysts could be operated up to 120 h TOS at remarkably high activity and stability. Productivity levels of up to 280 g_oligomers_ g_Ni_^−1^ h^−1^ could be demonstrated with the SCALMS system showing only minor deactivation when working under optimized conditions at 533 K. XPS and TEM measurements in combination with DFT calculations indicate the presence of Ga−Ni intermetallic phases and isolated Ni atoms that both are considered as active species for the observed catalytic properties. DFT/MD studies further emphasize the role and importance of hydrogen in generating catalytically active species. The observed mild loss in activity over time can be attributed to the depletion of Ni available for catalysis due to the formation of high melting Ga−Ni intermetallic phases happening at 623 K, as corroborated by XPS and TEM results. Similar phenomena have been observed in Ga−Pd and Ga−Rh systems suggesting intermetallic compound formation as a general deactivation mechanism of Ga‐based SCALMS systems.

## Supporting Information Summary

Additional results and experimental procedures are included in the Supporting Information (SI). The authors have cited additional references within the Supporting Information.[^50^, ^51^, ^52^, ^53^, ^54^, ^55^, ^56^, ^57^, ^58^, ^59^, ^60^, ^61^, ^62^, ^63^, ^64^, ^65^, ^66^, ^67^]

## Conflict of Interests

The authors declare no conflict of interest.

1

## Supporting information

As a service to our authors and readers, this journal provides supporting information supplied by the authors. Such materials are peer reviewed and may be re‐organized for online delivery, but are not copy‐edited or typeset. Technical support issues arising from supporting information (other than missing files) should be addressed to the authors.

Supporting Information

## Data Availability

The data that support the findings of this study are available from the corresponding author upon reasonable request.
