# Supplementary material for: Single Atom Sites in Ga‐Ni Supported Catalytically Active Liquid Metal Solutions (SCALMS) for Selective Ethylene Oligomerization
Source: Chemphyschem. 2025 Mar 14;26(10):e202400651. doi: 10.1002/cphc.202400651 (PMC12091850; doi:10.1002/cphc.202400651)
Supplement: Supplementary file 1 — Supporting Information [file CPHC-26-e202400651-s001.pdf]

ChemPhysChem

Supporting Information

Single Atom Sites in Ga-Ni Supported Catalytically Active Liquid Metal Solutions (SCALMS) for Selective Ethylene Oligomerization

Alexander Søgaard, Tzung-En Hsieh, Julien Steffen, Simon Carl, Mingjian Wu, Yousuf R. Ramzi, Sven Maisel, Johannes Will, Anna Efimenko, Mihaela Gorgoi, Regan G. Wilks, Johannes Frisch, Nicola Taccardi,* Marco Haumann,* Erdmann Spiecker, Andreas Görling, Marcus Bär, and Peter Wasserscheid*

Electronic Supporting Information (ESI)

Single Atom Sites in Ga-Ni Supported Catalytically Active Liquid Metal Solutions (SCALMS) for Selective Ethylene Oligomerization

Alexander Søgaard,[†a,b] Tzung-En Hsieh,[†c] Julien Steffen,[d] Mingjian Wu,[e] Simon Carl,[e] Yousuf Raed Ramzi,[a] Sven Maisel,[d] Johannes Will,[e] Anna Efimenko,[c] Mihaela Gorgoi,[c] Regan G. Wilks,[c] Johannes Frisch,[c] Nicola Taccardi,*[a] Marco Haumann,*[a,f] Erdmann Spiecker,[e] Andreas Görling,[d] Marcus Bär,[c,g,h] Peter Wasserscheid*[a,i]

a) Friedrich-Alexander-Universität Erlangen-Nürnberg (FAU), Lehrstuhl für Chemische Reaktionstechnik (CRT), Egerlandstr. 3, 91058 Erlangen, Germany.

b) CHEC Research Centre, Department of Chemical and Biochemical Engineering, Technical University of Denmark (DTU), Søltofts Plads 229, 2800 Kongens Lyngby, Denmark.

c) Helmholtz-Zentrum Berlin für Materialien und Energie GmbH (HZB), Department of Interface Design, Albert-Einstein-Str. 15, 12489 Berlin, Germany.

d) Friedrich-Alexander-Universität Erlangen-Nürnberg (FAU), Lehrstuhl für Theoretische Chemie, Egerlandstr. 3, 91058 Erlangen, Germany.

e) Institute of Micro- and Nanostructure Research & Center for Nanoanalysis and Electron Microscopy (CENEM), University of Erlangen-Nuremberg, IZNF, Cauerstr. 3, 91058 Erlangen, Germany.

f) Research Centre for Synthesis and Catalysis, Department of Chemistry, University of Johannesburg, P.O. Box 524, Auckland Park 2006, South Africa

g) Helmholtz-Institute Erlangen-Nürnberg for Renewable Energy (HI ERN), Department X-ray spectroscopy at interfaces of thin films, Albert-Einstein-Str. 15, 12489 Berlin, Germany.

h) Friedrich-Alexander-Universität Erlangen-Nürnberg (FAU), Department of Chemistry and Pharmacy, Egerlandstr. 3, 91058 Erlangen, Germany.

i) Forschungszentrum Jülich, Helmholtz-Institute Erlangen-Nürnberg for Renewable Energies (IEK 11), Egerlandstr. 3, 91058 Erlangen, Germany.

† These authors contributed equally to this work.*Corresponding author. e-mail address: N.T.: nicola.taccardi@fau.de; M.H.: marco.haumann@fau.de; P.W.: peter.wasserscheid@fau.de;

Experiment and Calculation Methods

Preparation Ga-Ni SCALMS catalysts for catalytic testing

The Ga-Ni SCALMS catalyst used for catalytic experiments, we synthesised in accordance with the procedure reported by our group.[1,2] The catalysts were prepared by a physical impregnation method[3,4] using ultrasonication (Branson 450 D sonifier) of Ga (0) to form a Ga emulsion in an alcoholic solvent. A 6 mm Ga nugget (99.9999%, Alfa Aesar) was dispersed in 100 mL of propan-2-ol using ultrasonication at at 80% intensity (ca. 80 W) for 30 min. The temperature was kept below 50 °C to avoid excessive oxidation of Ga. The Ni precursor, nickel (II) chloride ethylene glycol dimethyl ether complex (98%, Sigma Aldrich) was added to the Ga-propan-2-ol emulsion according to the targeted Ga-to-Ni atomic ratio. The support material, µ-spherical SiO_2 (Ø 75-200 µm, Sigma-Aldrich), was added to the Ga-Ni solution, targeting a final 7 wt% Ga loading of the catalyst. The support was impregnated *via* tumble drying at ca. 70 mbar and 40 °C until dry. The catalyst was calcined at 500 °C in air overnight and stored in ambient conditions until utilisation.

Metal content analysis of Ga-Ni SCALMS by ICP-AES

The Ga and Ni loading and the corresponding Ga:Ni-ratios of the SCALMS were determined by inductively coupled plasma atomic emission spectroscopy (ICP-AES) using a Ciros CCD (Spectro Analytical Instruments GmbH). The solid samples were digested in 3:1:1 volumetric ratio of concentrated $HCl:HNO_3:HF$ using microwave heating up to 220 °C for 40 min. The instrument was calibrated for Ni (231.604 nm) and Ga (417.206 nm) with standard solutions of the specific elements prior to the analyses.

Catalytic oligomerization of ethylene

The catalytic experiments were conducted in a continuously operated tubular fixed reactor. The product gas was analysed using on-line gas chromatography (GC) (Agilent 7890A with a 30 m, 0.32 mm GasPro® column) with a flame ionisation detector (FID).

For the temperature variation experiment, 1.0 g of $Ga_{67}Ni/SiO_2$ SCALMS catalyst was added to the reactor and fixed between glass wool plugs. The reactor was inertized under He and brought to

the initial reaction conditions (473 K and 0.6 MPa). Diluted ethylene (20 vol% C_2H_4 in He) was supplied at 50 mL_N min^{-1} into the reactor during ethylene oligomerisation for 36 h TOS. The temperature was then stepwise increased to 523, 573 and 623 K, each with a hold time of 36 h TOS. Lastly, the temperature was decreased to 523 K and held for a final 12 h TOS.

For the periodic purging experiments, the 1.0 g of Ga-Ni SCALMS catalyst ($Ga_{67}Ni/SiO_2$ or $Ga_{70}Ni/SiO_2$ for H_2/He and He purging, respectively) was added to the reactor and the setup was inertized with He. H_2 pretreatment of the catalyst was carried out for 2 h time on stream (TOS) at 583 K and atmospheric pressure (0.1 MPa) under undiluted H_2 (20 mL_N min^{-1}). The reactor was again inertized under He and brought to the initial reaction conditions (533 K and 0.6 MPa). Diluted ethylene (20 vol% C_2H_4 in He) was supplied at 50 mL_N min^{-1} into the reactor during ethylene oligomerisation for 20 h TOS followed by 4 h TOS of purging. This cycle was carried out for 5 cycles, bringing the total TOS to 120 h. The purging steps was carried out with either 100 vol% He (200 mL_N min^{-1}) for 4 h TOS or with 1 h TOS of 100 vol% He (200 mL_N min^{-1}), 2 h TOS of 20 vol% H_2 in He (100 mL_N min^{-1}) and finally 1 h TOS of 100 vol% He (200 mL_N min^{-1}), according the to the specific experiment. Details on the data treatment and calculations of the conversion, selectivity and productivity during experiments are provided in the ESI.

Sample preparation for synchrotron-based X-ray photoelectron spectroscopy (XPS)

All Ga-Ni alloy samples studied by XPS were prepared by physical vapor deposition (PVD) in an ultra-high vacuum (UHV) chamber attached to a UHV backbone (base pressure = $5x10^{-9}$ mbar) in the Energy Materials *In-Situ* Laboratory Berlin (EMIL).[5] The individually prepared Ga-Ni samples were directly transferred in UHV to the analysis chamber (→ SISSY-1, see below) for immediate synchrotron-based XPS/HAXPES analysis. A SPECS EBE-4 e-beam evaporator was utilized for sample preparation. All samples were prepared under vacuum conditions (base pressure $< 1 \times 10^{-8}$ mbar) by co-deposition of Ga (powder, 99.9999%, Sigma Aldrich) and Ni (rods, 99.98%, Sigma Aldrich) on a silicon wafer (Boron doped, Czochralsky tech. prepared, 2-4 Ω·cm resistance) having a native silicon oxide surface layer. The desired Ni concentration (100, 50, 20, 5, 2 at%) was determined by using deposition rates derived by using a quartz microbalance for the pure metals before alloy deposition. The Ni concentrations of different Ga-Ni alloys were determined

by fitting the XPS peaks and calculating the atomic ratio by considering photoelectronic cross section (σ)[6,7] and IMFP[8–10] (**Fig. S2 and S3**).

Sample Oxidation and Reduction Treatments:

In addition to the PVD setup, the preparation chamber also integrates an annealing stage, enabling sample annealing experiments up to 973K in partial pressures of oxygen (99.998%, Air Liquide) and hydrogen (99.999%, Air Liquide) that allows for, e.g. sample surface oxidation and reduction treatments, respectively. *In situ* annealing and in-system oxidation/reduction experiments were carried out with a nominal 2 at% Ni Ga-Ni sample (1.3 at% Ni examined by XPS analysis) in the following order. After characterization of the as prepared sample at 298K (as-prepared), the sample was *in situ* annealed to 673K and measured at same temperature (liquefied). The sample was then cooled to 298K (solidified). At each of these temperatures/states the sample was studied by XPS (*vide infra*). The surface oxidation and reduction are conducted in the UHV chamber for sample preparation ex-situ. The sample was oxidized at 298K in 1×10^{-3} Pa O_2 flow for 30 mins and studied by photoemission at 298K (oxidized). After the oxidation test, the sample was reduced at 573K in 1×10^{-3} Pa H_2 flow for 30 mins and studied by photoemission at 533K (H_2 reduced). Sample transfer in UHV ($< 5\times10^{-8}$ Pa) was ensured.

Synchrotron-based Photoelectron Spectroscopy Measurements:

Soft/hard x-ray photoelectron spectroscopy (XPS/HAXPES) experiments of the as-prepared and differently treated Ga-Ni samples were conducted in the SISSY-1 endstation fully exploiting EMIL's two-color beamline, providing soft X-ray radiation from an UE48-PGM undulator/monochromator combination and hard X-ray radiation from an U17-DCM setup – located at the BESSY II, which was operated by the Helmholtz-Zentrum Berlin für Materialien und Energie GmbH (HZB). The soft and hard X-rays was focused on the same spot on the sample in the SISSY-1 endstation providing photons in an energy range between 80 eV and 10.000 eV. The SISSY-1 endstation (at a base pressure $< 2\cdot10^{-9}$ mbar) was equipped with a Scienta EW 4000 hemispherical electron analyzer allowing XPS and HAXPES measurements.

Ga 2p, Ni 2p core levels and the region of the valence band (VB) were measured using a pass energy of 20 eV and photon energies resulting in two different kinetic photoelectron energies (KE = 100 eV and 500 eV) to derive (depth-dependent) information from the surface and near-surface bulk region of the sample. The survey spectra were measured using a pass energy of 50 eV with

620 eV excitation energy. In addition, HAXPES measurements with a pass energy of 100 eV were performed with a photon energy (hν) of 4900 eV, to probe the bulk-related properties of the sample. The inelastic mean free path (IMFP) of the photoelectrons with KE = 100 eV and 500 eV in the studied sample was 0.5 and 1.1 nm, respectively, and the photoelectron IMFP of the hν = 4900 eV measurements varies (depending on the resulting E_K) between 5.0 and 5.3 nm.[8–11]

Transmission Election Microscopy (TEM) Analysis

(Scanning) transmission electron microscopy (S/TEM) studies were performed using a ThermoFisher Scientific (TFS) Titan Themis microscope operated at 300 kV. The samples were prepared by PVD (as described for the XPS studies) on DENSsolution wildfire chips, which are MEMS-based chips enabling rapid and precise heating of sample region of interest up to 1000 ^{o}C within seconds. The chips are inserted in a DENSsolutions single tilt heating holder and inserted in the TEM for *in situ* heating observations. The TEM was equipped with C_s-correctors both at the illumination and imaging side, along with a Super-X energy dispersive X-ray Spectroscopy system (EDX). In the STEM imaging and SI experiments, the probe-forming semi-angle was set to a value of 15.7 mrad. Probe current of ~60 pA and dwell time 10 – 20 µs were applied for simultaneous high-angle annual dark-field (HAADF), ADF, annual bright-field (ABF) and BF STEM imaging; and increase probe current 100 - 150 pA and dwell time 30–50 µs was used for STEM-EDX mapping. The pixel sampling size was set to 0.5 – 1 nm, depending on the set probe current, to balance sufficient signal-to-noise ratio and spatial resolution while avoiding beam damage of the samples. EDX signals are accumulated with multi-frame data acquisition. The total acquisition time were between 10 – 15 min before obvious beam effect was observed. The EDX data were analyzed using TFS Velox software and quantified using standard k-factor method. In HRTEM imaging, the screen current was set to 2 - 4 nA and images were captured at 3 fps in continuous mode, or 0.33s exposure time for each frame. Selected area electron diffraction (SAED) data were acquired using SA aperture including a round area with diameter of about 3.4 µm, providing a statistical relevant number of particles.

DFT calculations and MD simulations

Spin-polarized density-functional theory (DFT) calculations were performed using the VASP (Vienna Ab Initio Simulation Package) code, applying the projector augmented wave (PAW) method to represent the atomic cores. The valence electrons were described with a plane wave

basis set, the cutoff was set to 250 eV if only Ga and Ni were included and to 300 eV, if hydrogen was involved as well.[12–14] The exchange-correlation functional developed by Perdew, Burke and Ernzerhof (PBE) was applied to describe exchange-correlation effects.[15] The energy levels at the Fermi level were broadened with a first-order Methfessel-Paxton smearing with a width of 0.2 eV[16] in the case of random alloy simulations and with Gaussian smearing with a width of 0.04 eV in the case of machine-learning (ML) dynamics.

The convergence criterion for the electronic SCF cycle was set to 10^{-7} eV. In geometry optimizations, lattice constants and atomic positions were relaxed with a force convergence criterion of $5\cdot10^{-3}$ eV/A and only the Γ-point was sampled.

Bader charges were calculated using all-electron charge densities as obtained from VASP.[17,18] Furthermore, larger 4x4x4 k point meshes were used for electronic density of states (DOS) and charge calculations in combination with a tetrahedron smearing with Blöchl corrections.[19]

Machine learning force fields (ML-FF) as implemented in the recent VASP version[20–22] were generated for the $Ga_{67}Ni$ alloy and its surface. For this, a slab cell (13.5 x 13.5 x 43 Å) containing 201 Ga and 3 Ni atoms was generated with a simple cubic geometry and randomly placed Ni atoms. The learning was performed during a NVT *ab initio* molecular dynamics (AIMD) simulation of 40.000 time steps (10 fs) length in total at a temperature of 533 K, simulated by the Nose-Hoover thermostat, where the *ab initio* forces were gradually replaced by the forces from machine learning, until more than 5000 time steps in a row were propagated with the ML-FF (similar settings as in ref. [21]). The cutoffs for the two- and three-body descriptors were set to 8 and 6 Å, respectively.

With the generated ML-FFs, slightly larger surface slabs of (13.5 x 13.5 x 67.8 Å) containing 335 Ga and 5 Ni atoms were sampled with MD. First, ten trajectories of 2.000.000 time steps (5 fs) each were sampled starting from different preequilibrated random slabs at 533 K, the results were averaged. Secondly, five trajectories of 2.000.000-time steps each (2 fs) were averaged for simulation temperatures of 300, 400, 500, 600 and 700 K, using a ML-FF trained at 700 K, respectively.

AIMD trajectories were sampled to study the adsorption behaviour of hydrogen atoms and molecules on Ni atoms near the surface. For those, smaller surface slabs as for the machine learning, containing 201 Ga and 3 Ni atoms, were first sampled with the ML-FF for 2 ns with a time step of 2 fs in three independent trajectories. From those, structures with Ni atoms at the

surface were picked; four AIMD trajectories were started with no hydrogen atoms, one hydrogen atom attached to the Ni near the surface, and a hydrogen molecule attached to the Ni near the surface. All trajectories were sampled for 40 ps with a time step of 1 fs.

Catalytic oligomerization of ethylene

Calculations for catalytic performance assessment

The following equations were used to evaluate the catalytic performance of the SCALMS catalysts.The **conversion** (X_{ethylene}) is calculated according to the following equation:

$$X_{ethylene} = \frac{n_{ethylene,0} - n_{ethylene}}{n_{ethlyene,0}} = 1 - \frac{n_{ethylene}}{n_{ethylene,0}} \qquad \text{Eq. 1}$$

where $n_{ethylene}$ is the molar amount of ethylene in the gas stream and $n_{ethylene,0}$ is the initial amount of ethylene in the gas stream.

The **selectivity** of product alkene *i* is expressed by the following equation :

$$S_i = \frac{n_i - n_{i,0}}{n_{ethylene,0} - n_{ethylene}} \cdot \frac{|\nu_{ethylene}|}{\nu_i} \qquad \text{Eq. 2}$$

where $n_{k,0}$ and $n_{i,0}$ represent the initial number of moles of substrate and product, respectively, and n_k *and* n_i represent the final number of moles of substrate and product, respectively.

The Ni-based productivity of the catalyst is calculated according to the following equation:

$$P_{oligomers} = \frac{\dot{n}_{ethylene} \cdot X_{ethylene}}{m_{Ni}} \cdot \sum_i S_i \cdot M_i \cdot \frac{\nu_i}{|\nu_{ethylene}|} \qquad \text{Eq. 3}$$

Where $\dot{n}_{ethylene}$ is the molar flow of ethylene in mol h^{-1}, m_{Ni} is the mass of Ni and M_i is the molar mass of oligomer *i*.

Scanning electron microscopy with energy-dispersive X-ray spectroscopy (SEM-EDX)

All analyses were carried out using a Phenom™ XL from Thermo Fisher Scientific. The electron beam was set to 15 kV at map intensity and high vacuum (1 Pa) while detecting the back-scattered electrons.

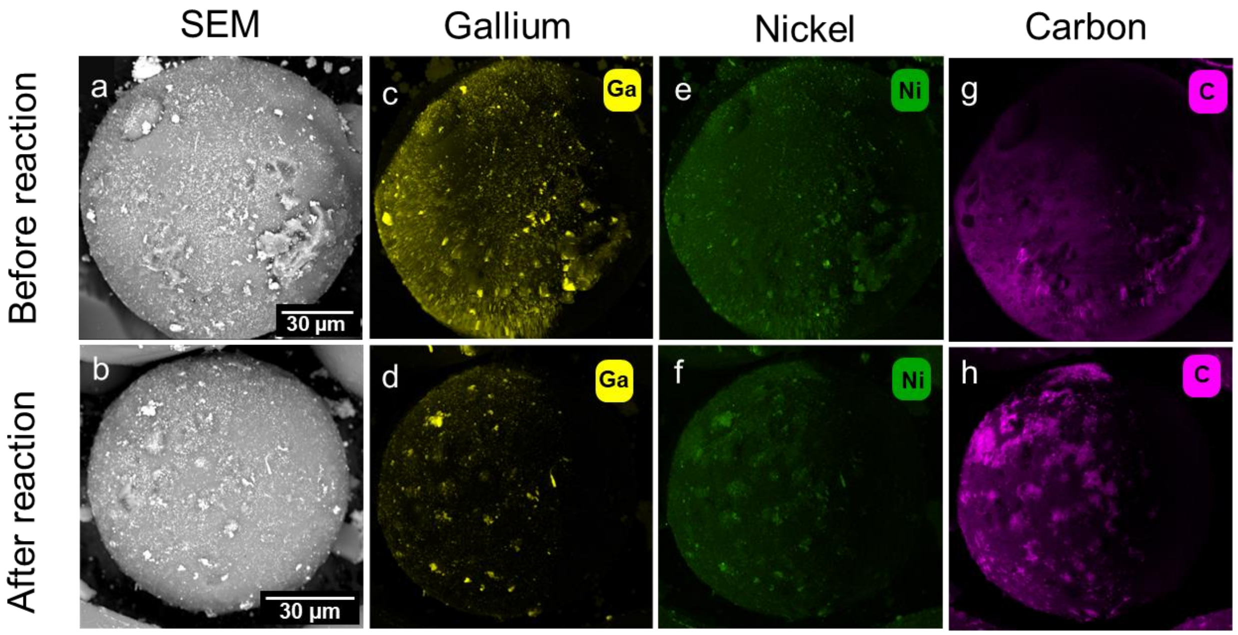

Figure S1: Images of $Ga_{67}Ni/SiO_2$ before (a,c,e,g) and after (b,d,f,h) reaction of ethylene oligomerisation presented in Fig.1. Scanning electron microscopy images (a,b) show the dispersion of Ga-Ni droplets on the spherical SiO_2 particle. Scale bars on image (= 30 µm). (c,d) EDX maps of Ga in yellow before and after reaction (c and d, respectively). (e,f) EDX maps of Ni in green before and after reaction (e and f, respectively). (g,h) EDX of C in purple before and after reaction.

Table S1: Elemental composition and catalytic performance of the catalysts presented in the main manuscript.

Entry	Catalyst ID	Ga[a] / wt%	Ni[a] / wt%	Ga:Ni-ratio / $mol_{Ga}\ mol_{Ni}^{-1}$	Productivity[b,c] / $g_{oligomer}\ g_{Ni}^{-1}\ h^{-1}$	
					C4	total
1	TN743	3.34	0.04	67	210	225
2	YR14	7.59	0.082	75	209	244
3	AS037	3.79	0.065	49	242	278

[a] determined by ICP-AES

[b] reaction conditions: 533 K, 0.6 MPa, 1.0 $g_{catalyst}$, 3000 $mL_N\ g_{catalyst}^{-1}\ h^{-1}$ (GHSV = 490 h^{-1}), 20 vol% C_2H_4 in He. Pretreatment conditions: 2 h time on stream (TOS), 583 K, 0.1 MPa, 1200 $mL_N\ g_{catalyst}\ h^{-1}$, 100 vol% H_2.

[c] productivity values obtained after 1.5 h time on stream (TOS)

Synchrotron-based photoemission spectroscopy (XPS)

Determination of GaOX film thickness

In this study, a simple overlayer model is utilized to discuss the $Ga_2O_{3-\delta}$ layer formation on top of metallic Ga, assuming a mechanism of homogeneous, closed packed oxide film growth.[23–25] The following equation can be used to calculate the GaO_X film thickness, D:

$$D = \lambda_{i,\mathrm{GaO_x}} \cdot \ln\left[\frac{I_{i,\mathrm{GaO_x}} \cdot \lambda_{i,\mathrm{Ga}} \cdot \mathrm{N(Ga)_{Ga}}}{I_{i,Ga} \cdot \lambda_{i,\mathrm{GaO_x}} \cdot \mathrm{N(Ga)_{GaO_x}}} + 1\right] \qquad \text{Eq. 4}$$

$\lambda_{i,GaOx}$ and $\lambda_{i,Ga}$ are the IMFP values in GaO_X and metallic Ga, respectively for core level *I* (calculated using the TPP2-M equation with the density and electron configuration of stoichiometric Ga_2O_3 as the absorbing layer – we considered this the best approximation available due to the lack of reliable parameters for $Ga_2O_{3-\delta}$). The $\lambda_{i,GaOx}$ and $\lambda_{i,Ga}$ of Ga 3d photoelectrons are 20.9 and 27.2 Å; of Ga 3p photoelectrons are 19.7 and 25.6 Å; of Ga 2p photoelectrons are 5.3 and 6.3 Å, respectively. $I_{i,GaOx}$ and $I_{i,Ga}$ are the intensities (i.e., areas) of the $Ga_2O_{3-\delta}$ and Ga peak contributions, respectively, derived for the core level *i* (obtained by XPS data fitting, see **Fig. S5** and S6). $N(Ga)_{GaOx}$ and $N(Ga)_{Ga}$ are the atomic densities of Ga in Ga_2O_3 (0.038 Atoms per cubic Å) and Ga (0.053 Atoms per cubic Å), respectively.23 It's noted that the formula for D assumes a uniform, closed capping oxide layer, and thus the discrepancies in the oxide thicknesses calculated using Ga 2p (**Table S3**) and using Ga 3d (**Table S4**) are related to the observed incomplete coverage of the Ga, which will cause an underestimation of the layer thickness in both cases, with the effect being more pronounced for the more surface sensitive data. As the oxide coverage of Ga increases, the results of the two calculations converge, with the remaining disagreement possibly attributable to the influence of morphology – i.e., the differing relative surface/bulk contributions of the nanoparticles compared to the smooth layer assumed in the calculation.

Quantification of Ni concentration

We quantitatively analyzed the Ni concentration during all steps of surface manipulation and as a function of the kinetic energy of the photoemitted electrons with different probing depth. The fitting results of Ga 2p and Ni 2p peaks in combination with photoelectron cross-sections (σ)[6,7] and the inelastic mean free path (IMFP),[8–11] can be used to examine the concentration of Ni in GaNi alloys using equations 5 and 6:

$$\frac{Ni}{Ga} = \left(\frac{I_{Ni}}{\sigma_{Ni}} \div \frac{I_{Ga}}{\sigma_{Ga}} \cdot \frac{\mathrm{IMFP}_{Ga}}{\mathrm{IMFP}_{Ni}}\right) \qquad \text{Eq. 5}$$

$$[Ni] = \left(\frac{\left(\frac{Ni}{Ga}\right)}{1 + \left(\frac{Ni}{Ga}\right)}\right) \cdot 100\ at\ \% \qquad \text{Eq. 6}$$

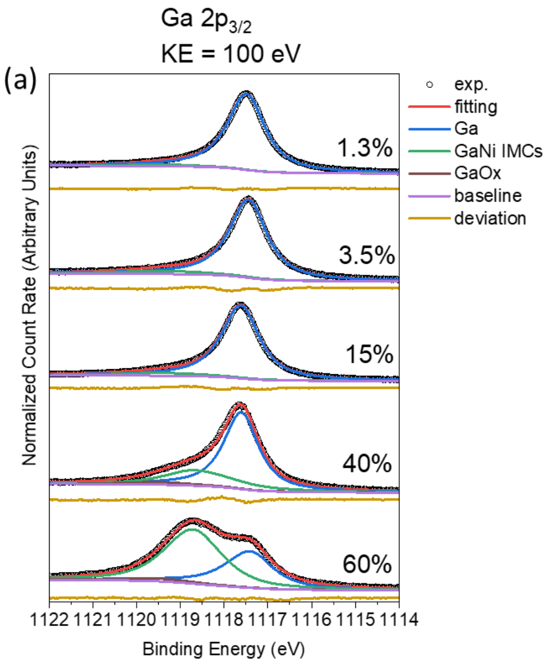

Figure S2: Fitting results of Ga $2p_{3/2}$ and Ni $2p_{3/2}$ core level peaks of Ga-Ni alloys with different Ni concentrations (at%). The probing photon energies were chosen such that the kinetic energy (KE) of photoelectrons were fixed at 100 eV.

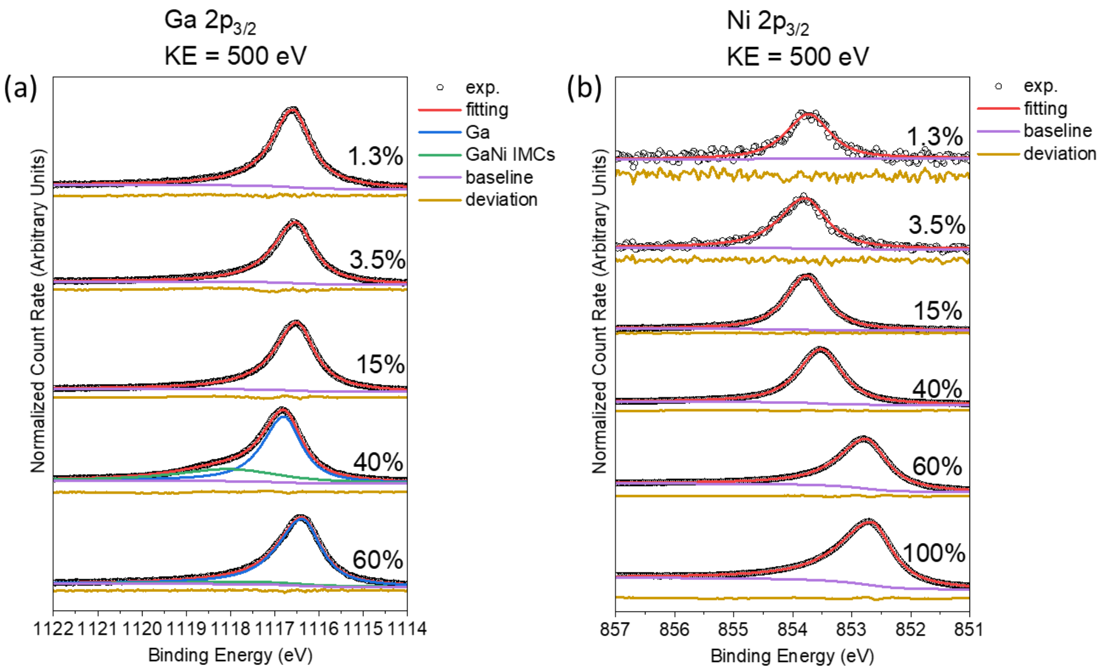

Figure S3: Fitting results of Ga $2p_{3/2}$ and Ni $2p_{3/2}$ core level peaks of Ga-Ni alloys with different Ni concentrations (at%). The probing photon energies were chosen such that the kinetic energy (KE) of photoelectrons were fixed at 500 eV.

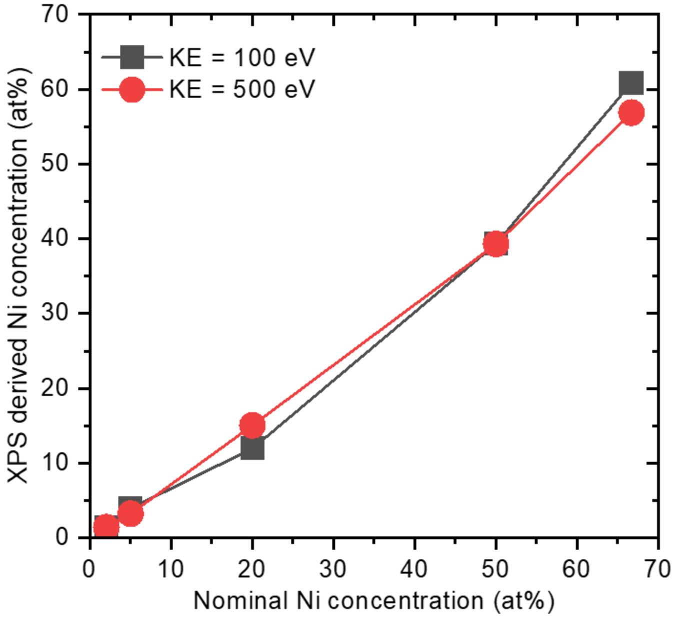

Figure S4: Comparison of nominal Ni concentration examined by quartz microbalance and concentration examined via XPS data having different KE (shown in **Fig. S2** and **S3**.

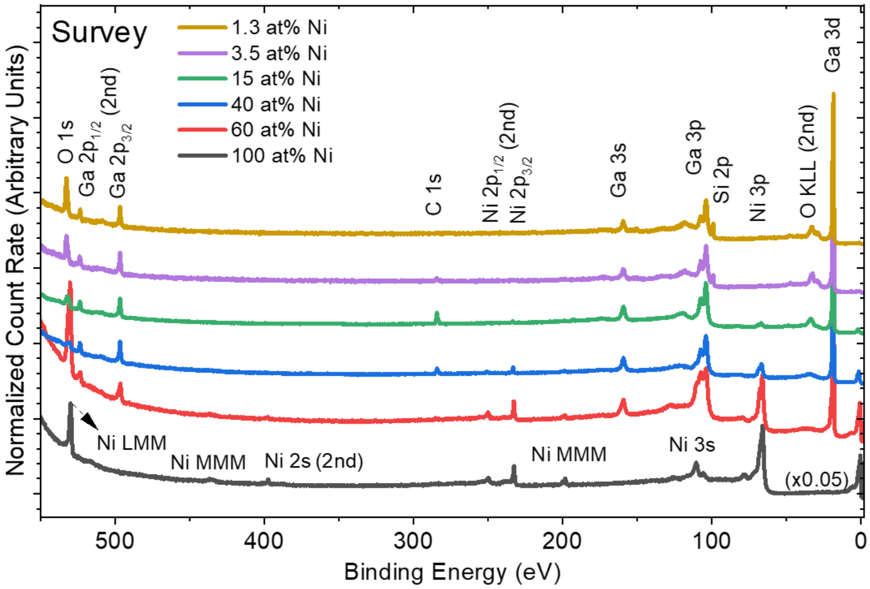

Figure S5: XPS survey spectra of PVD-deposited Ga-Ni alloys with different Ni concentrations in at %. The spectra were measured using a photon energy of 620 eV. The Ga 2p and Ni 2p core level peaks are recorded because of their excitation by X-rays coming from the second harmonic of the monochromator and are thus indicated with a "2nd" tag.

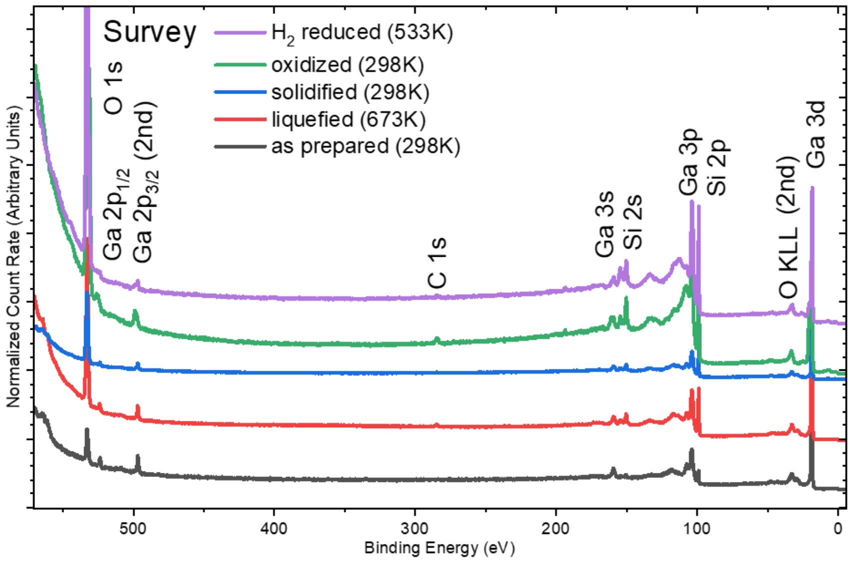

Figure S6: XPS survey spectra of a 1.3% Ni contained Ga-Ni alloy measured at different temperatures / after different sequential post-deposition treatments: as-prepared (black), liquefied (673K, red), solidified (i.e. after annealing and cool down to room temperature, blue), oxidized (green), and H_2 reduced (H_2-assisted reduction, purple). Measurement temperatures are listed in legend. The spectra are measured with 620 eV of photon energy.

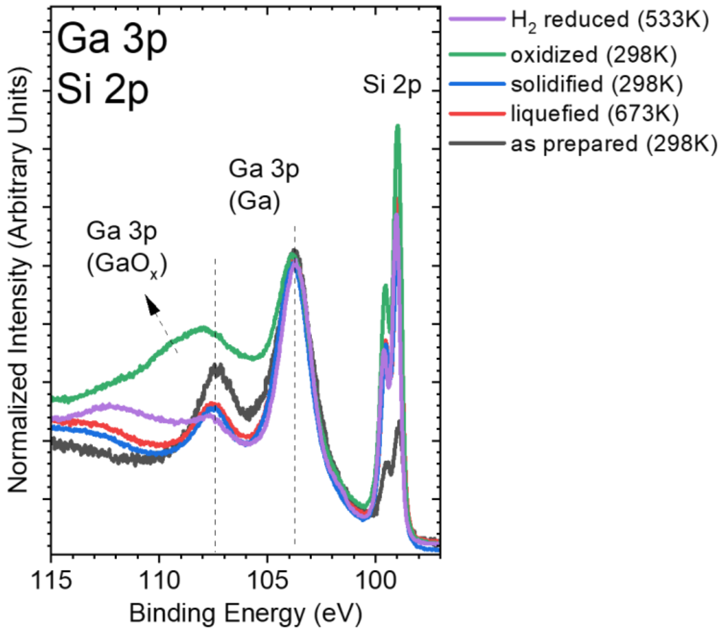

Figure S7: Overlapped Ga 3p and Si 2p spectra of PVD-deposited Ga-Ni alloys with 1.3 at% Ni at different temperatures / after different sequential after post-treatments.

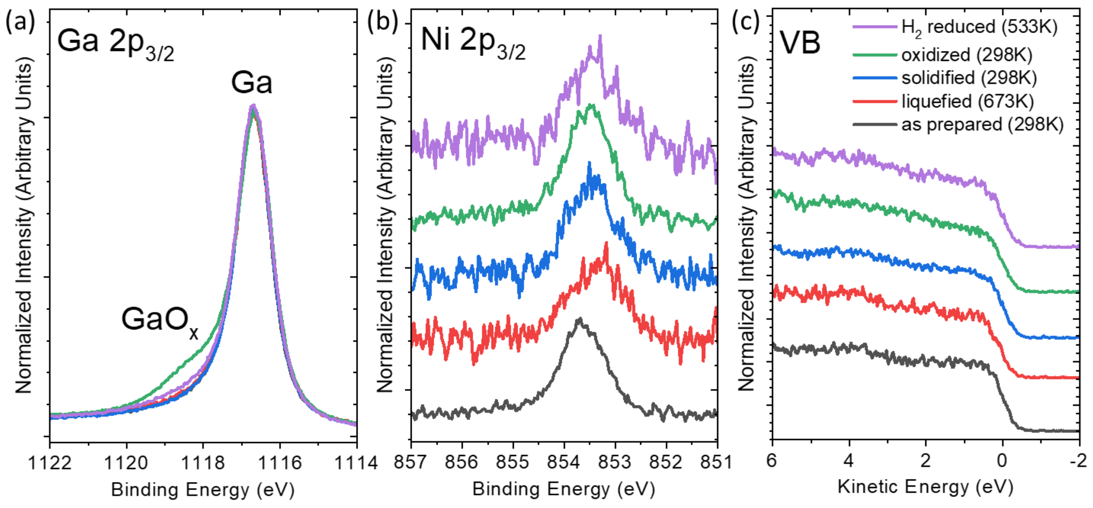

Figure S8: (a) Ga $2p_{3/2}$, (b) Ni $2p_{3/2}$ and (c) valence band HAXPES spectra of PVD-deposited Ga-Ni alloys with 1.3 at% Ni after post-treatments. The photon energy is set to 4900 eV for all HAXPES measurements.

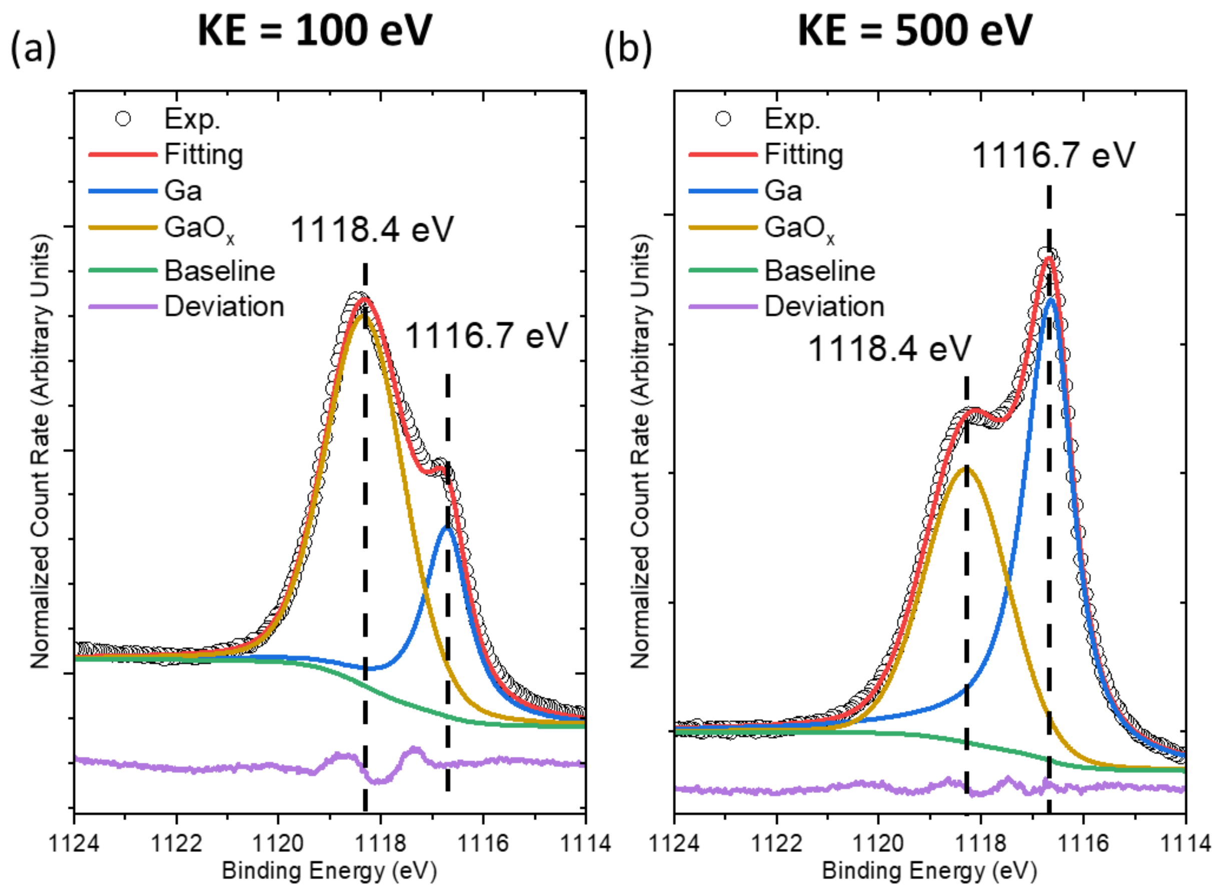

Figure S9: Fitting results of Ga $2p_{3/2}$ of 1.3 at% Ni Ga-Ni alloy measured with (a) KE = 100eV and (b) KE = 500eV. The sample is oxidized under $1x10^{-3}$ Pa O_2 for 30 mins.

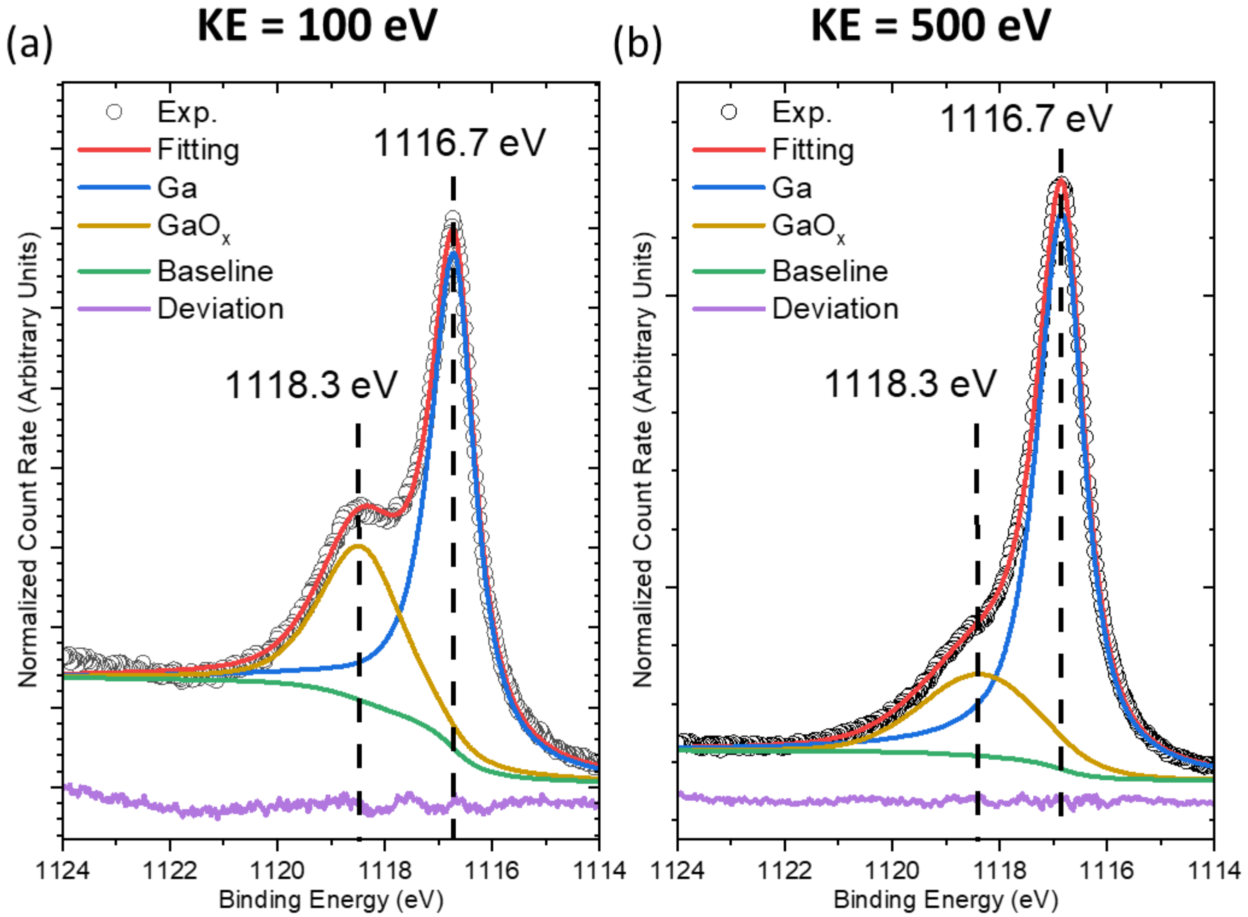

Figure S10: Fitting results of $Ga2p_{3/2}$ of 1.3 at% Ni Ga-Ni alloy measured with (a) KE = 100eV and (b) KE = 500eV. The sample was reduced under $1x10^{-3}$ Pa H_2 for 30 mins.

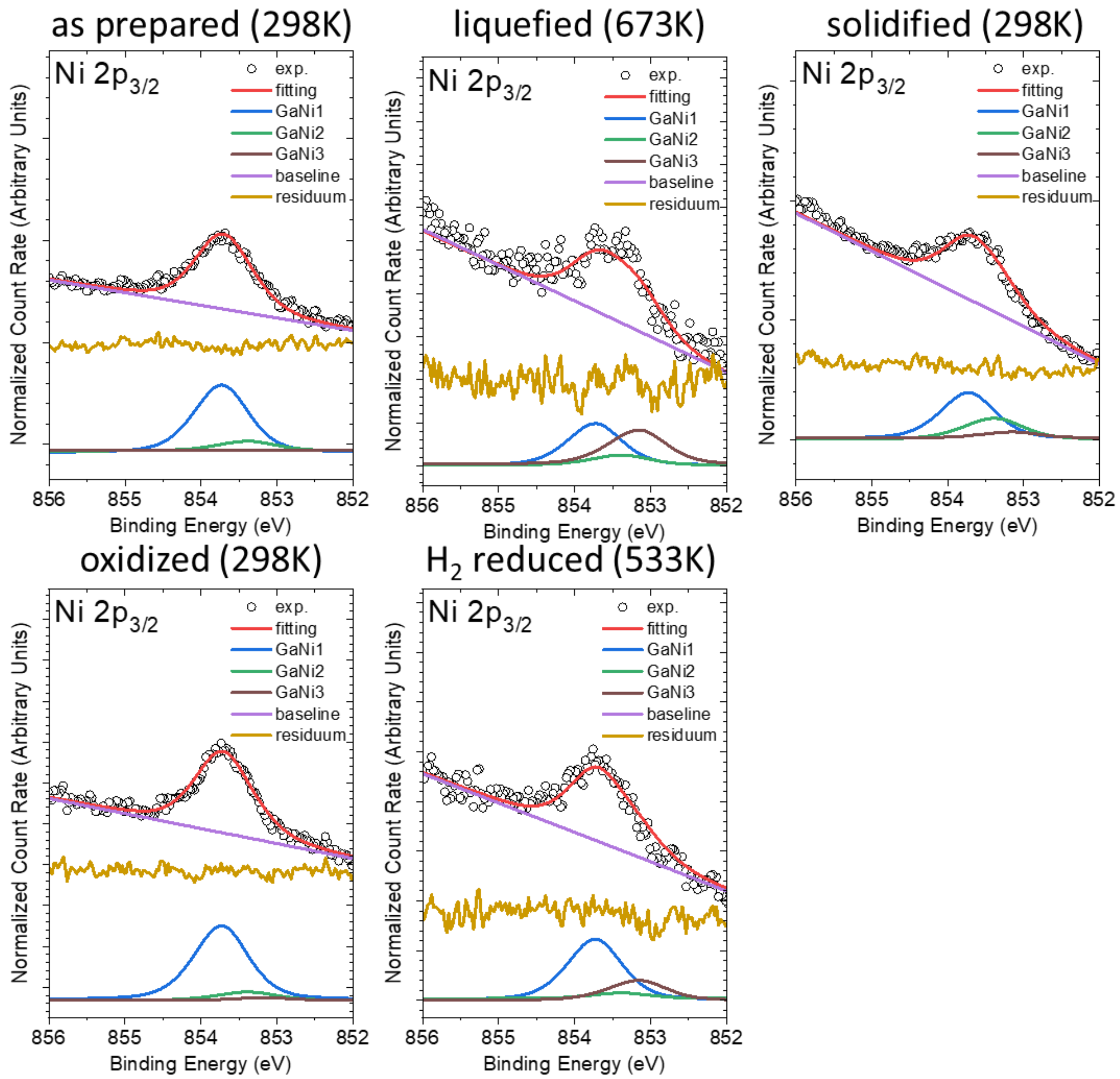

Figure S11: Fitting results of surface sensitive (KE = 100 eV) Ni $2p_{3/2}$ spectra of PVD-deposited Ga-Ni alloys with 1.3 at% Ni at different temperatures/after different sequential post-deposition treatments. The spectra are fitted with 3 features: GaNi1 denotes to random alloy, GaNi2 denotes to Ga-Ni intermetallic phases formed at room temperature, GaNi3 denotes to Ga-Ni intermetallic phases forming around 573K. This speciation is done according to the results of the microscopic investigations.

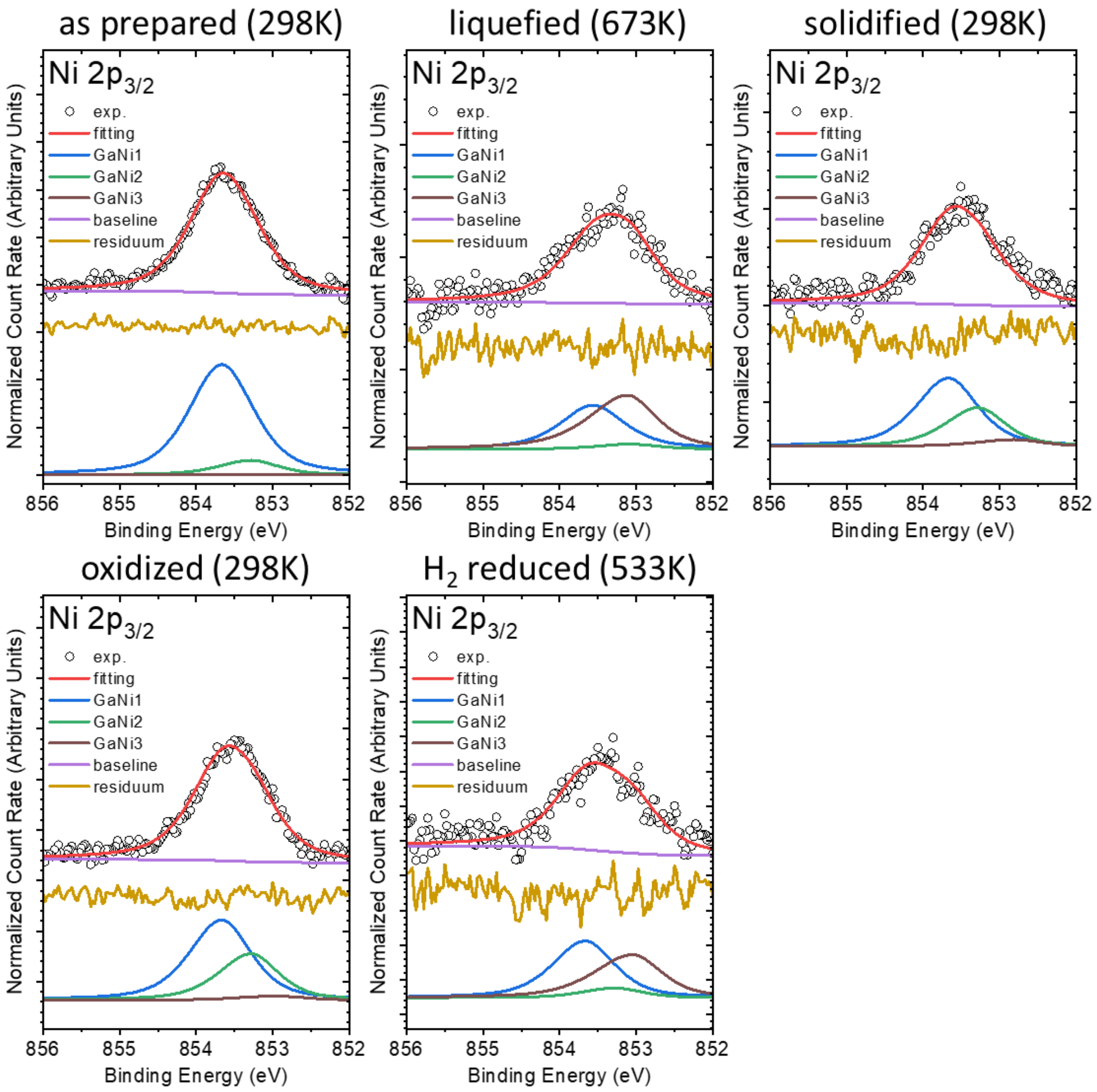

Figure S12: Fitting results of bulk sensitive Ni $2p_{3/2}$ spectra (HAXPES, photon energy=4900 eV) of PVD-deposited Ga-Ni alloys with 1.3 at% Ni at different temperatures/after different sequential post-deposition treatments. The spectra are fitted with 3 features: GaNi1 denotes to random alloy, GaNi2 denotes to Ga-Ni intermetallic phases formed at room temperature, GaNi3 denotes to Ga-Ni intermetallic phases forming around 573K. This speciation is done according to the results of the microscopic investigations.

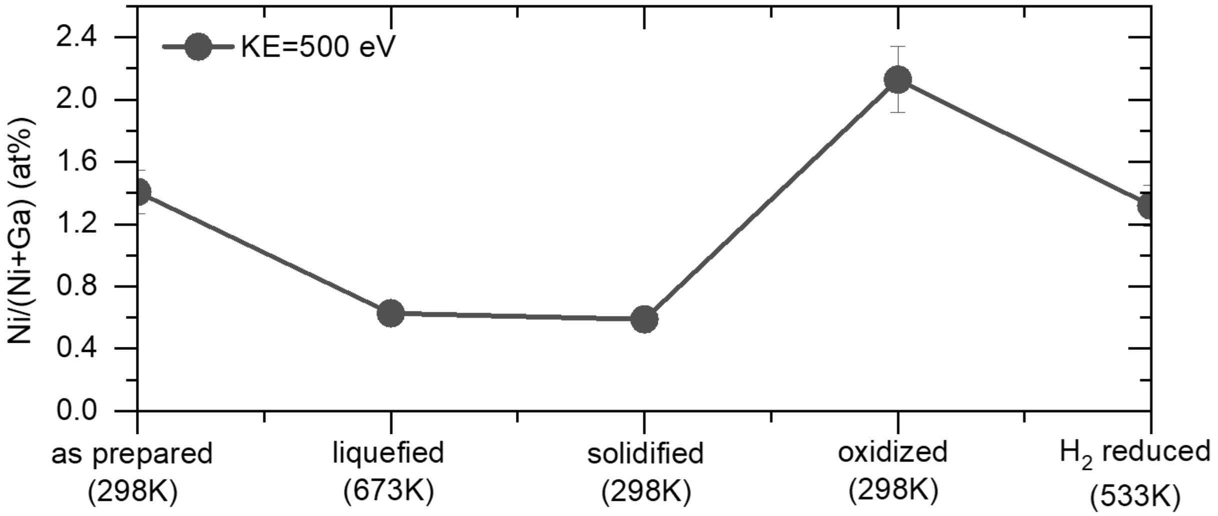

Figure S13: Evolution of the XPS derived Ni concentration in the Ga-Ni sample for different temperatures/at different post-deposition treatment steps measured for kinetic energies = 500 eV (that correspond to IMFP values of 1.1 nm) of the detected Ga $2p_{3/2}$ and Ni $2p_{3/2}$ photoelectrons.

Table S2. XPS derived Ni concentration of the studied PVD-deposited Ga-Ni sample series based on the KE=100 eV data set. The IMFP value is calculated by the TPP-2M formula.[8–11] Photoionization cross section (σ) is referred to the tabulated value from Trzhaskovskaya, Nefedov, and Yarzhemski.[6,7]

KE = 100eV			
Sample	Core level	Peak area (a.u.)	[Ni] (at%)
$Ga_{49}Ni$	Ga $2p_{3/2}$	255161	**1.3**
	Ni $2p_{3/2}$	4389.10	
$Ga_{19}Ni$	Ga $2p_{3/2}$	114318	**3.9**
	Ni $2p_{3/2}$	5966.15	
Ga_4Ni	Ga $2p_{3/2}$	266784	**12.0**
	Ni $2p_{3/2}$	46123.8	
GaNi	Ga $2p_{3/2}$	241576	**40.0**
	Ni $2p_{3/2}$	199289	
$GaNi_2$	Ga $2p_{3/2}$	165653	**61.1**
	Ni $2p_{3/2}$	327916	
σ of $Ga2p_{3/2}$ & $Ni2p_{3/2}$ = **4.94 & 6.29 e^{-3} (Å²)**<br>IMFP of Ga & Ni = **4.8 Å**			

Table S3. XPS derived Ni concentration of the studied PVD-deposited Ga-Ni sample series based on the KE=500 eV data set. The IMFP value is calculated by the TPP-2M formula.[8–11] Photoionization cross section (σ) is referred to the tabulated value from Trzhaskovskaya, Nefedov, and Yarzhemski [6,7].

KE = 500eV			
Sample	Core level	Intensity (a.u.)	[Ni] (at%)
$Ga_{49}Ni$	Ga $2p_{3/2}$	17590.2	**1.4**
	Ni $2p_{3/2}$	272.373	
$Ga_{19}Ni$	Ga $2p_{3/2}$	23725.7	**3.3**
	Ni $2p_{3/2}$	859.880	
Ga_4Ni	Ga $2p_{3/2}$	139391	**15.0**
	Ni $2p_{3/2}$	26767.6	
GaNi	Ga $2p_{3/2}$	99222.4	**39.1**
	Ni $2p_{3/2}$	69709.0	
$GaNi_2$	Ga $2p_{3/2}$	73920	**56.8**
	Ni $2p_{3/2}$	105747	
σ of $Ga2p_{3/2}$ & $Ni2p_{3/2}$ = **2.37 & 2.57 e^{-3} (Å²)**<br>IMFP of Ga & Ni = **10.8 Å**			

Table S4. GaO_x layer thickness derived from the synchrotron-based XPS measurements of $Ga2p_{3/2}$ core level with fixed kinetic energy to 100 eV and 500 eV.

Sample	Kinetic Energy (eV)	Species	Peak area (%)	GaO_x thickness (nm)
**Ga_{49}Ni_oxidized**	100 eV	Ga	34	**0.68±0.1**
		GaO_x	66	
	500 eV	Ga	64	**0.77±0.1**
		GaO_x	36	
**Ga_{49}Ni_H_2 reduced**	100 eV	Ga	24	**0.19±0.1**
		GaO_x	76	
	500 eV	Ga	87	**0.25±0.1**
		GaO_x	13	

Table S5. Ni/(Ni+Ga) ratio of $Ga_{49}Ni$ at different temperatures/after different treatment steps. The results are examined via synchrotron-based XPS measurements with fixed photoelectron kinetic energies (100 eV and 500 eV) and HAXPES measurements with fixed photon energy of 4900 eV (KE of Ni $2p_{3/2}$ and Ga $2p_{3/2}$ is equal to 4057 and 3733 eV, respectively) for the probed Ga and Ni core levels. The IMFP value is calculated by the TPP-2M formula.[8–11] Photoionization cross section (σ) is calculated by the tabulated value from Trzhaskovskaya, Nefedov, and Yarzhemski.[6,7]

Treatment step	Kinetic Energy (eV)	Core level	Peak intensity (a.u.)	Ni/(Ni+Ga) (at%)
**As prepared (298K)**	100 eV	Ni $2p_{3/2}$	4389.1	**1.3**
		Ga $2p_{3/2}$	255160	
	500 eV	Ni $2p_{3/2}$	272.4	**1.4**
		Ga $2p_{3/2}$	17590	
	4057 eV	Ni $2p_{3/2}$	1601.2	**1.2**
	3733 eV	Ga $2p_{3/2}$	208380	
**Liquefied (673K)**	100 eV	Ni $2p_{3/2}$	637.9	**0.5**
		Ga $2p_{3/2}$	101713	
	500 eV	Ni $2p_{3/2}$	412.3	**0.6**
		Ga $2p_{3/2}$	51243	
	4057 eV	Ni $2p_{3/2}$	208.3	**0.5**
	3733 eV	Ga $2p_{3/2}$	70556	
**Solidified (298K)**	100 eV	Ni $2p_{3/2}$	3167.5	**0.6**
		Ga $2p_{3/2}$	426525	
	500 eV	Ni $2p_{3/2}$	582.9	**0.6**
		Ga $2p_{3/2}$	77347	
	4057 eV	Ni $2p_{3/2}$	291.0	**0.4**
	3733 eV	Ga $2p_{3/2}$	116811	
**Oxidized (298K)**	100 eV	Ni $2p_{3/2}$	2567.3	**1.8**
		Ga $2p_{3/2}$	110031	
	500 eV	Ni $2p_{3/2}$	1571.5	**2.1**
		Ga $2p_{3/2}$	56803	
	4057 eV	Ni $2p_{3/2}$	582.3	**0.8**
	3733 eV	Ga $2p_{3/2}$	116910	
**H_2 reduced (533K)**	100 eV	Ni $2p_{3/2}$	1205.0	**1.5**
		Ga $2p_{3/2}$	62375	
	500 eV	Ni $2p_{3/2}$	745.6	**1.4**
		Ga $2p_{3/2}$	43830	
	4057 eV	Ni $2p_{3/2}$	360.7	**0.6**
	3733 eV	Ga $2p_{3/2}$	96745	
**σ of Ga2$p_{3/2}$ & Ni2$p_{3/2}$ = 4.94 & 6.29 e^{-3}Å^2 (100 eV); 2.37 & 2.57 e^{-3}Å^2 (500 eV); 0.09 & 0.06 e^{-3}Å^2 (3733/4057eV)<br>IMFP of Ga & Ni = 4.8 Å (100 eV); 10.8 Å (500 eV); 50.5/53.3Å (3733/4057 eV)**				

Transmission Electron Microscopy (TEM)

Movie S1: file 1624 in situ SAED observation from t2 till + 304s (total of 912 frame recorded at 3fps, avi playback at 20fps, or ~7x accelerated)

Movie S2: file 1640 in situ HRTEM observation at 550C from t5 + 111s (total of 333 frame recorded at 3fps, avi playback at 20fps, or ~7x accelerated)

Movie S3: file 1615, in situ bright field TEM observation from t0 - t1 according to time markers in Fig. S16. (total of 703 frames recorded at 3fps, avi playback at 20fps, or ~7x accelerated)

The movies are provided via cloud storage using the following link:

https://faubox.rrze.uni-erlangen.de/getlink/fi2GPtHjNUBhGoeFgRgUkW/

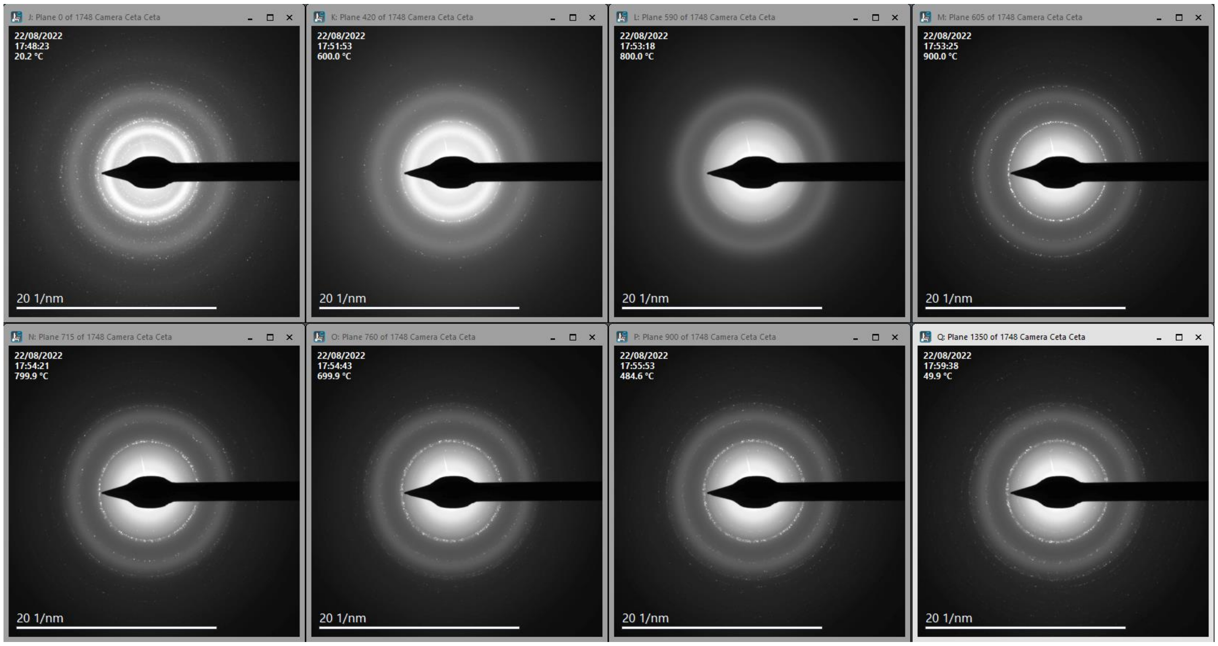

Figure S14: In situ SAED experiments up to 900 C. The formation of Ga-Ni phase took place at the cost of almost total displacement of liquid Ga, as suggested by the disappearance of narrow halo ring at around 4.2 nm^{-1}.

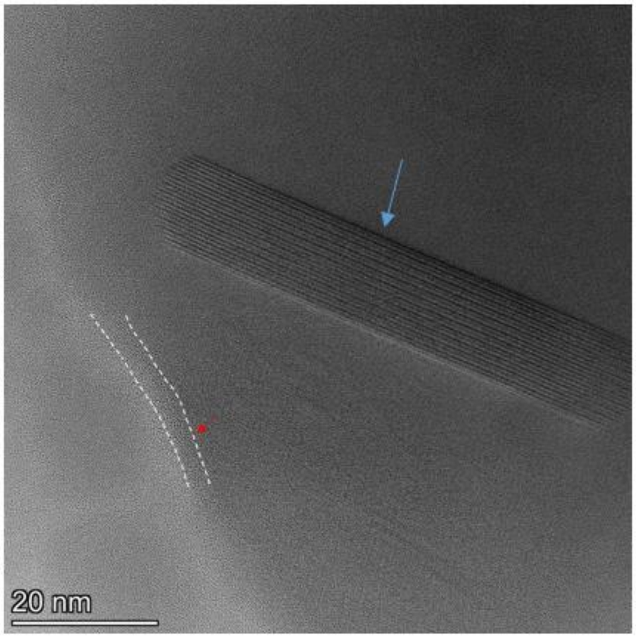

Figure S15: BF HRTEM image of the as-deposited sample. The ~2-3 nm oxide shell could be seen by the slight contrast of the different amorphous-like contrast at the edge of the particle (marked by the double white dash lines & red arrow). The lattice contrast of crystalline Ga-Ni intermetallic phase is confined *within* the oxide shell (blue arrow).

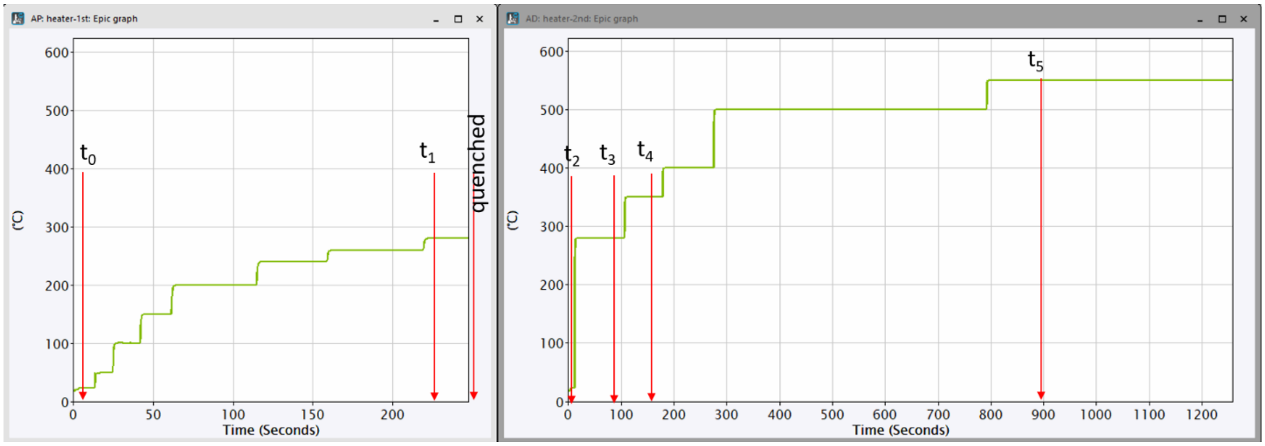

Figure S16: Temperature profiles applied during two successive *in situ* observation (t_0 and t_1 in imaging- and t_2 to t_5 in diffraction-mode). At the end of the first heating session, the heating device is switched off, so the sample is quenched down to 298 K in a couple of seconds. Time markers indicate the images/diffraction patterns shown in **Fig. 5** in the main text.

DFT calculations and MD simulations

Ga-Ni random alloys

The initial geometries for Ga-Ni random alloys were constructed by randomly replacing a certain number of Ni atoms in a fcc-Ni bulk cell (14.07 x 14.07 x 14.07 Å) containing 256 atoms by Ga (10 to 90 at% Ga in steps of 10 at.%.). After geometry optimization of all alloys, different properties were computed for them, i.e., Bader charges, core level binding energies and energies of states.

The center of the Ni 3d band ε_d was derived from the partial DOS and evaluated as

$$\epsilon_d = \frac{\int x\rho(x)\,dx}{\int \rho(x)dx} \qquad \textbf{Eq. 7}$$

where $\rho(x)$ is the electronic DOS distribution of the Ni 3d band along energy x.

For all Ni atoms Ni 2p core level (CL) binding energies were computed in initial state (IS) and final state (FS) approximation as described in detail in ref. 18. Here a 2x2x2 k point mesh was found to be sufficient to yield converged binding energies. As the screening of the core electrons is not contained in PAW calculations, errors in the absolute binding energies appear. Therefore, core level shifts (CLS) with respect to pure Ni bulk are calculated.

For the Ga-Ni random alloys with different Ni concentrations, the magnetic moments and the optimized lattice constants are shown in **Table S6**. The size of the shell shrinks with growing Ni concentration; further, the magnetic moment is zero for concentrations below 60 at.% and small for 70 at.%, in agreement with the plotted spin densities in the main manuscript (**Fig. 6**).

Table S6. Magnetic moments per unit cell and lattice constant for all unit cells with different Ni concentration at the PBE level.

Entry	Ni concentration / at%	Magnetic moment per unit cell	Optimized lattice constant / Å
1	10	0	16.74
2	20	0	16.36
3	30	0	15.83
4	40	0	15.45
5	50	0	15.12
6	60	0	14.66
7	70	1.56	14.48
8	80	33.44	14.29
9	90	104.80	14.17
10	100	182.71	14.07

Ga-Ni Surface Slab Machine Learning Simulations

The time-dependent z-coordinate of one of the Ni atoms in a Ga-Ni surface slab simulated with ML-FF-MD at 533 K is shown in **Fig. S17**. The Ni moves twice from one edge of the slab to the other within 8 ns, mainly staying roughly 3 to 5 Å below the surface (corresponding to the Ni density maximum shown in **Fig. 6c** in the main manuscript). For short time intervals, however, it directly approaches the gas-liquid interface (shown on the right). Three screenshots of the surface region were added to give a better impression of the actual atom distribution in those time intervals.

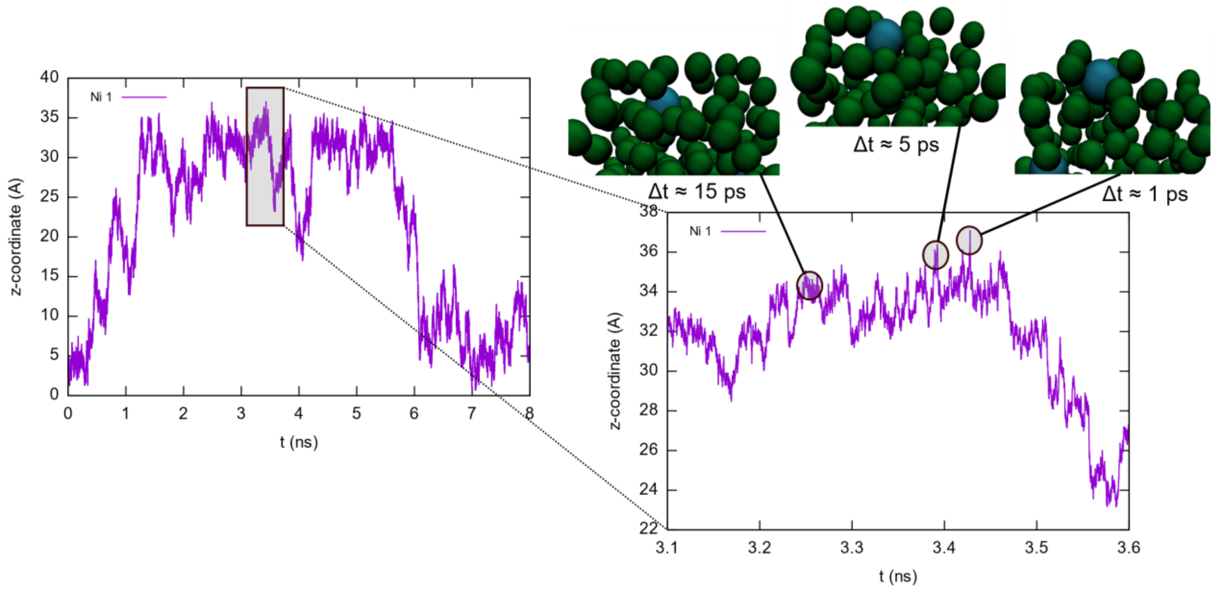

Figure S17: Time dependent position of a Ni atom taken from one of the ML-FF trajectories of a Ga-Ni surface slab at 533 K. Shown are screenshots of the structures where a Ni approaches the surface of the slab for some ps (estimated retention time Δt given in ps).

Ga and Ni element densities along the surface normal, generated by 50 ns of ML-FF simulations for each system (analogous to **Fig. 6c** in the main manuscript), are shown in **Fig. S18**.

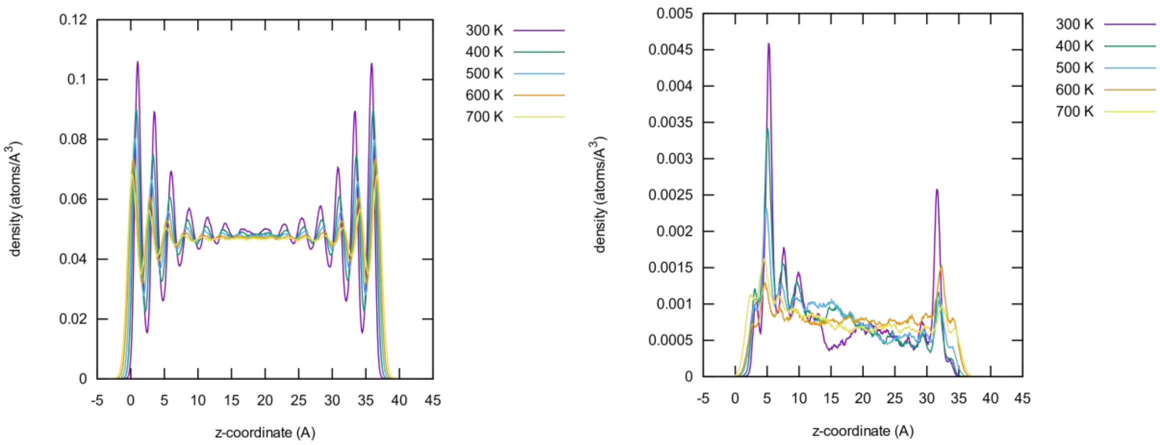

(a) Ga (b) Ni

Figure S18: Element densities of Ga and Ni with respect to the surface normal, obtained from ML-FF simulations at different temperatures, between 300 and 700 K.

With raising temperature, the density profile becomes less structured especially at the surface, where a strong layering can be seen for Ga at 300 and 400 K. The Ni atoms, on the other hand,

seem to remain a large part of the time near their initial positions for 300 and 400 K (and partly 500 K), where large maxima can be seen (this effect becomes even stronger for 100 and 200 K, not shown here due to large heights of maxima). At 600 and 600 K, on the other hand, the Ni profile becomes a lot smoother, indicating a less viscous state with better ability of Ni atoms to move around.

This trend, with a phase transition between 400 and 600 K, is also visible when investigating the probability of finding a Ni atom within the outer Ga surface layer (evaluated from the profiles in **Fig. S18**). This is shown in **Table S7**. The Ni surface probability is 0.5 % or less for 300 and 400 K and around 7 % for 600 and 700 K, with an intermediate value of 2 % at 500 K. It can thus be followed that the more liquid state at higher temperatures enhances the rate of Ni atoms appearing at the liquid-gas interface.

Table S7. Probability to find a Ni atom within the outer Ga surface layer in a surface cutout of 13.5 x 13.5 Å, calculated from ML-FF sampling trajectories of $Ga_{67}Ni$.

Entry	T / K	Ni probability / %
1	300	0.361
2	400	0.504
3	500	2.094
4	600	6.685
5	700	7.715

Ga-Ni Surface Slab Core Level Shift Calculations

In **Fig. S19**, core level shifts (averaged over 5 Ni atoms within independent systems each) of Ni 2p core levels are shown for different temperatures looked at in the simulations. Here, bulk region (10 Å or more below the liquid-gas interface) and surface region (less than 10 Å below that interface) are distinguished. In both cases, a negative shift of IS ad FS levels can be seen with raising temperature with no significant difference between bulk and surface regions.

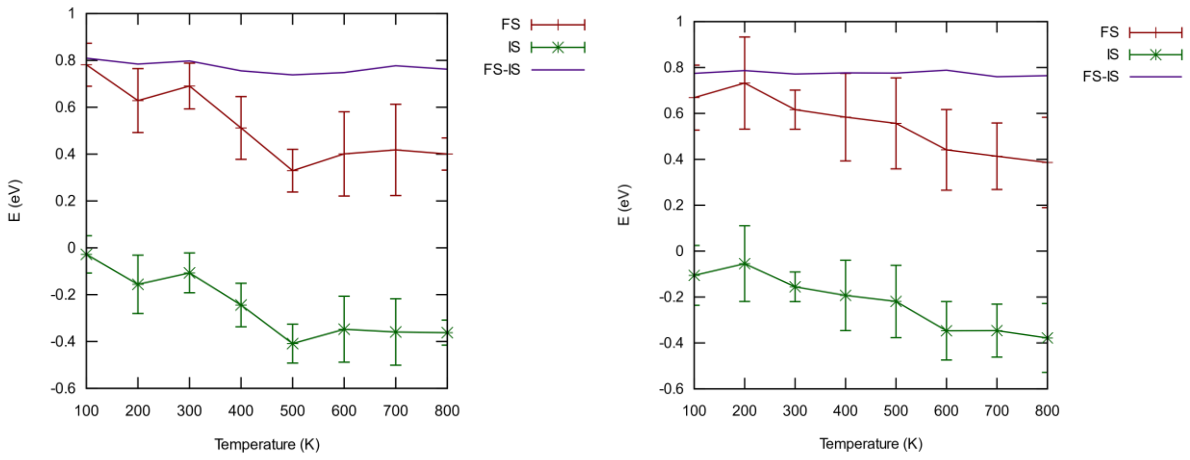

(a) bulk (b) surface

Figure S19: Temperature dependent IS and FS Ni 2p core level shifts calculated for ML-FF screenshots of surface slabs. Bulk means the Ni atom was located 10 Å or more below the surface and surface means the Ni atom is located less than 10 Å below the surface.

In **Fig. S20**, a similar analysis was done for the Ga-Ni surface slab at 533 K. The system was divided into slices along the z-axis, each slice having a width of roughly 0.5 Å. Structures were picked from the ML-FF trajectory with one of the Ni atoms located in the respective slice; this was done for 5 different ML-FF trajectories in order to get some averaging over local environments.

No clear trend with respect to IS and FS energy levels can be seen if trajectory screenshots are evaluated directly (a). It seems that the energies grow slightly when going to bulk regions (larger z values), but the change is below 0.1 eV and thus too small for robust conclusions. If the geometries are relaxed before calculating the CLS, on the other hand (corresponding to quick-freezing of the model), the trend becomes more clearly, with the energies of Ni atoms directly at the surface being 0.1 to 0.2 eV smaller than those in the bulk, while the final state effect (FS-IS) remains essentially constant.

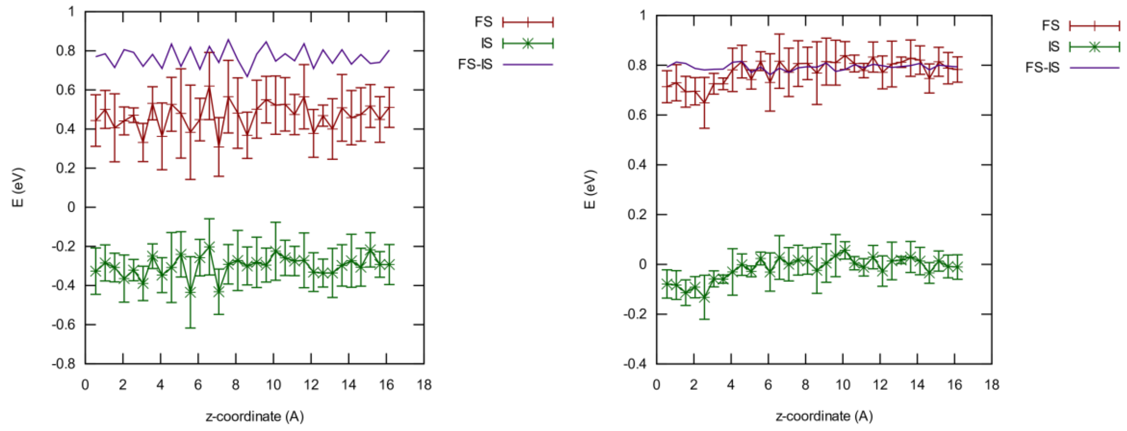

(a) MD screenshots (b) relaxed geometries

Figure S20: z-coordinate resolved Ni 2p IS and FS core level shifts taken from surface slab ML-FF-MD screenshots (a) or relaxed thereafter (b). The gas-liquid interface is located at z≈0 (see also Figure 1c in the main manuscript).

A similar analysis was done concerning the Bader charges of Ni atoms, now only with one calculation per slice (**Fig. S21**). No trend can be seen here for unrelaxed or relaxed snapshots, the magnitude of the Ni partial charge is always around -0.5, no matter if the atom is located at the surface or in the bulk. The example frames in (b) show that all Ga atoms build a positive background charge, and not just the direct solvation shell around the Ni atoms.

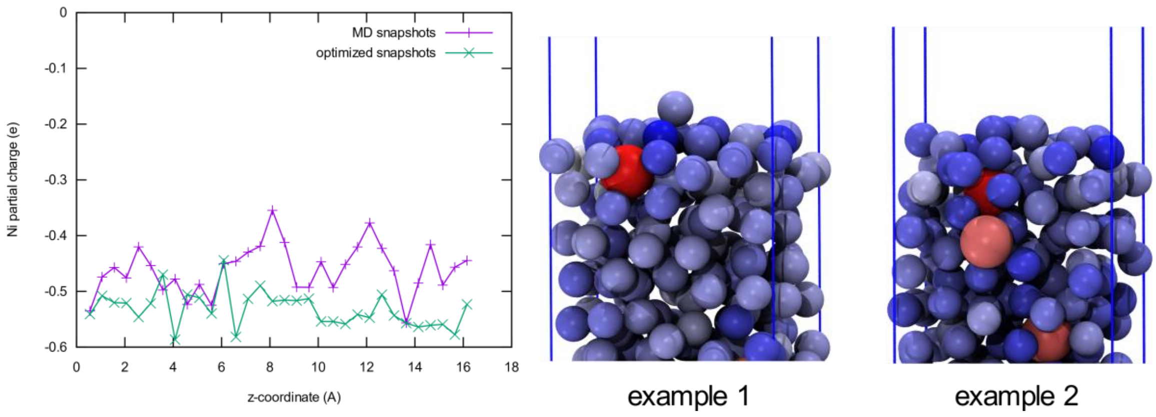

(a) Ni Bader charges in slab (b) example frames

Figure S21: z-coordinate resolved Ni Bader charges for surface slam ML-FF-MD screenshots (a) and two example charge distributions for two of the screenshots. Red means negative charge, blue means positive charge, grey means zero net charge. Ni atoms are shown larger than Ga atoms.

Hydrogen Adsorption on Ni Atoms

Here, additional results like those in Figure 1d in the main manuscript are shown. Four AIMD trajectories were calculated for systems with no H atom, one H atom and a H_2 molecule attached on a surface Ni atom, respectively. The initial setup was obtained by picking structures with one Ni atom located directly at the surface of the Ga-Ni slab from ML-FF trajectories at 533 K.

AIMD trajectories were started directly or after placing one or two H atoms on the “active” Ni atom (the trajectories without H could of course been simulated with ML-FF as well; to exclude any relative method error, however, all were calculated with DFT in this case).

In **Fig. S22**, example screenshots of four different trajectories are presented. The first two show the quick disappearance of Ni from the surface if no H is present. In (c) and (d) (also shown in Figure 1d in the main manuscript), the effect of one H to hold the Ni at the surface is visualized. Screenshots (e) and (f) show a hydrogen molecule placed on the Ni, which quickly desorbs into the gas phase. Finally, (g) and (h) also show a H_2 on the Ni, but now the H-H bond is split shortly after the beginning; one H stays at the Ni and holds it at the surface (as for the H_1 system), the second slowly diffuses from one Ga to another at the gas-liquid interface, serving as potential reservoir of active hydrogen for the case that another Ni appears at the surface.

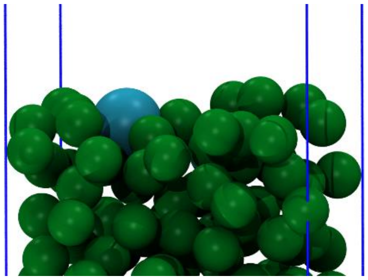

(a No H, 0 ps

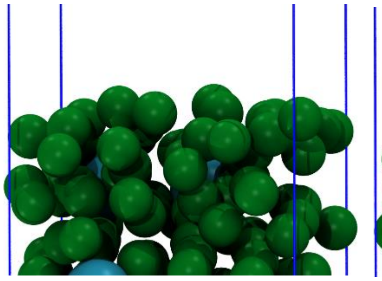

(b) no H, 10 ps

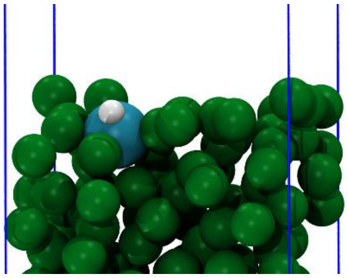

(c) H_1, 1 ps

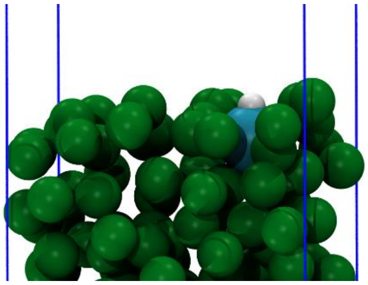

(d) H_1, 40 ps

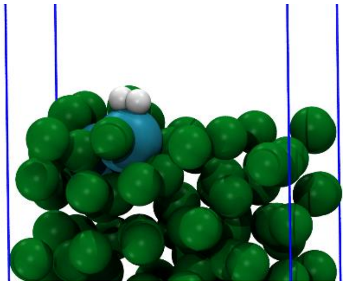

(e) H_2, case1, 0 ps

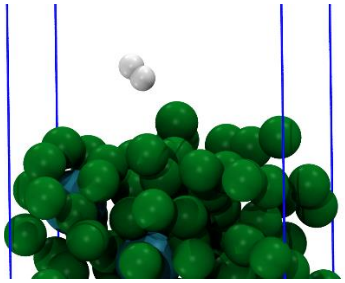

(f) H_2, case1, 10 ps

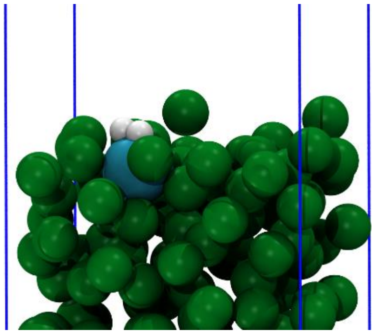

(g) H_2, case2, 0 ps

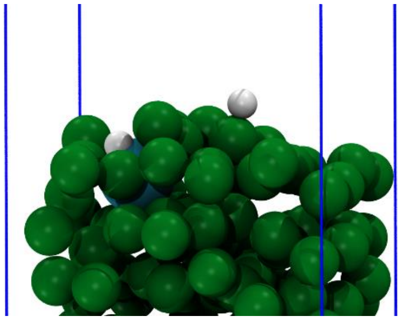

(h) H_2, case2, 15 ps

Figure S22: Screenshots of AIMD trajectories of Ga-Ni surface slabs with no, one or two H atoms placed on a Ni which is located at the surface at t=0. The screenshots relate to the plots in the Figures below as follows: No H (**Fig. S23**, trajectory 4), H_1 (**Fig. S24**, trajectory 4), H_2 case 1 (**Fig. S25**, trajectory 2), H_2 case 2 (**Fig. S25**, trajectory 1).

In **Fig. S23**, all four trajectories of systems starting with a Ni (always the Ni_3 atom in the plots, where Ni_1 and Ni_2 are located in the bulk slightly below the surface) at the surface are evaluated. The time-dependent z-coordinates of the atoms are shown as well as the density profile of Ga atoms resulting from averaging over the trajectory frames to give a better impression of the position relative to the surface.

In all four cases, the Ni atom located within the outer Ga layer quickly moves back into the bulk (in 5 to 10 ps), in good agreement with the ML-FF evaluation in **Fig. S18**. The atom, however, does not move deep into the bulk but stays within the second-outer Ga layer, where the “reservoir” of Ni atoms potentially taking place into the catalysis are located, in good agreement to the density profile shown in Figure 1c in the main manuscript.

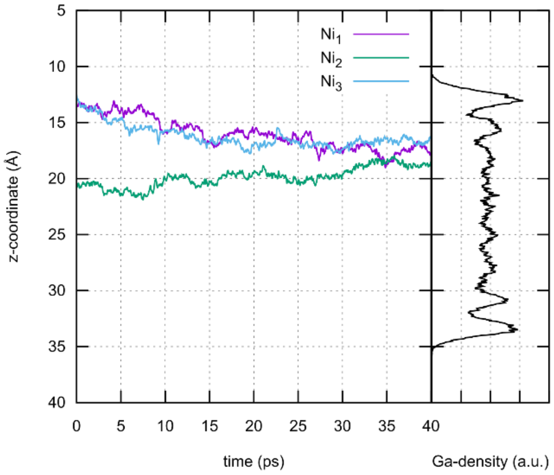

(a) trajectory 1

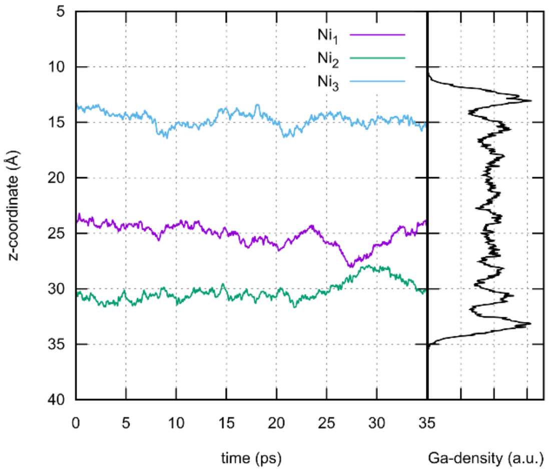

(b) trajectory 2

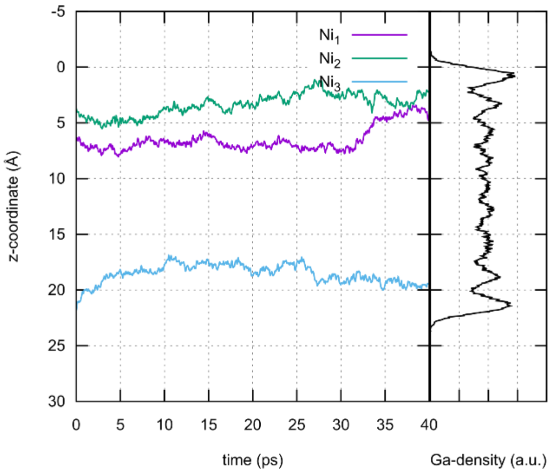

(c) trajectory 3

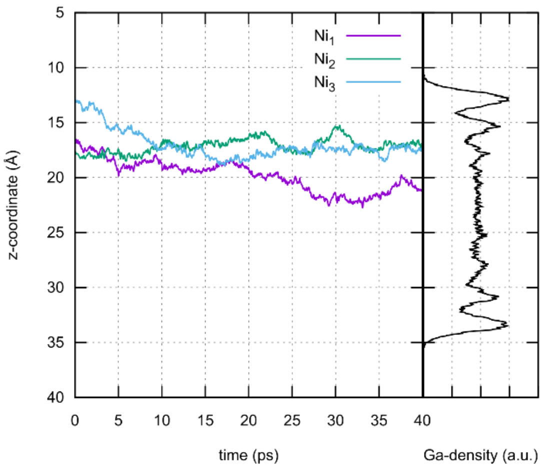

(d) trajectory 4

Figure S23: Time dependent z-coordinates of all Ni atoms obtained from AIMD simulations of Ga-Ni surface slabs with one Ni (Ni_3) initially placed at the surface and two others (Ni_1 and Ni_2) placed in the bulk region.

The trajectories with one H placed on the surface Ni atom (again Ni_3 in the plots) are shown in **Fig. S24**. All cases essentially show the same behaviour. The H atom stays above the Ni and holds it in the upper Ga layer and thus accessible to the gas phase.

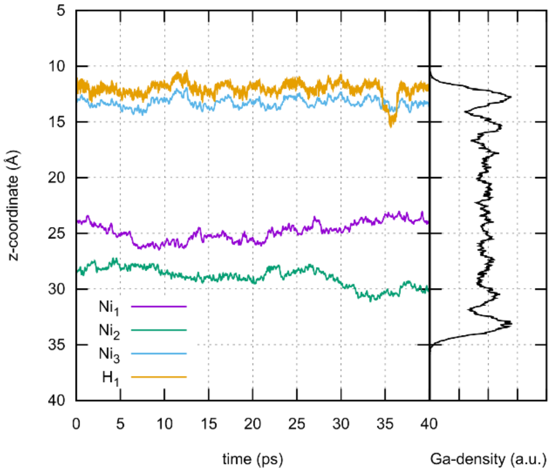

(a) trajectory 1

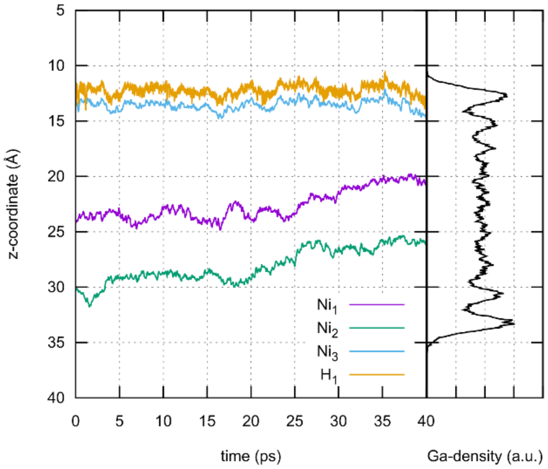

(b) trajectory 2

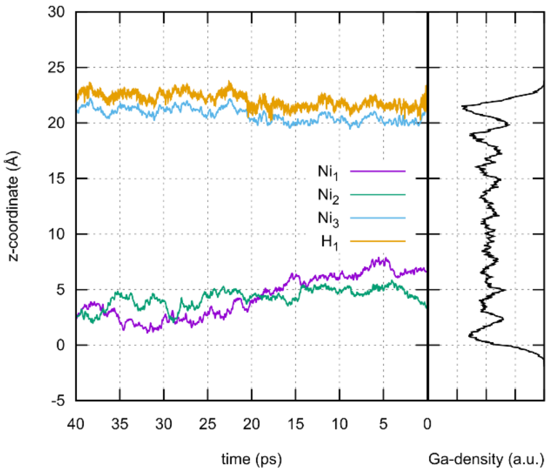

(c) trajectory 3

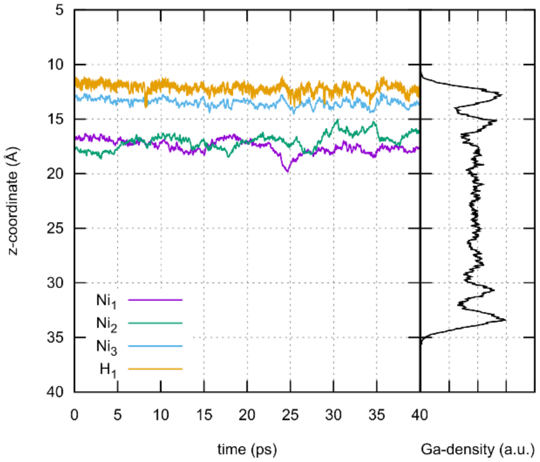

(d) trajectory 4

Figure S24: Time dependent z-coordinates of all Ni and H atoms obtained from AIMD simulations of Ga-Ni surface slabs with one Ni (Ni_3) initially placed at the surface with a H atom on it, and two other Ni atoms (Ni_1 and Ni_2) placed in the bulk region.

Finally, the trajectories of H_2 molecules placed on the surface Ni atom (again Ni_3 in the plots) are shown in **Fig. S24**. Here, two possible behaviours can be seen. In (a), the H_2 molecule gets split shortly after the beginning of the trajectory (see also **Fig. S22**, (e)-(f)). Both H atoms stay at the surface most of the time, where the first atom stays bound to the Ni and the second atom is slightly bound on different Ga atoms (located 1-2 Å above the first one). After 30 ps, the Ni-H dimer begins to move away from the surface to the bulk, into the second Ga layer. Longer simulations will be done in the future with ML-FFs covering Ga, Ni and H atoms (and other SCALMS systems) to

study the long-term behaviour. The second possibility, presumably the more probable one, can be seen in (b)-(d). The H molecule stays intact and quickly desorbs into the gas phase, where it moves back and forth between the edges of the slab, performing elastic collisions with the Ga surface (the Ni atoms go back into the second Ga layer). Due to reinitialization of velocities during the trajectories, which were calculated 15-20 parts to circumvent the wall-time limit of the calculation cluster, the H_2 molecules sometimes revert the movement directly in the gas phase.

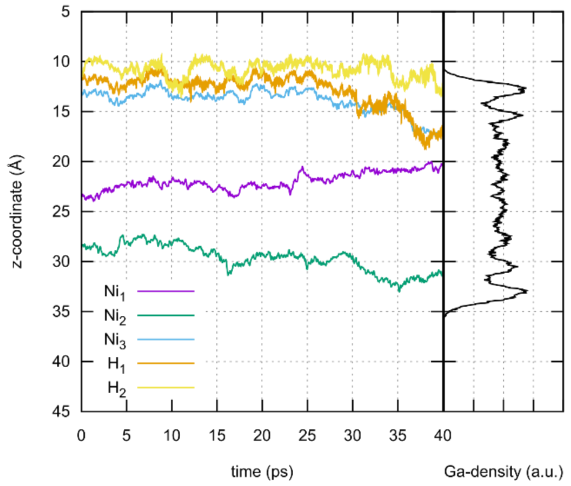

(a) trajectory 1

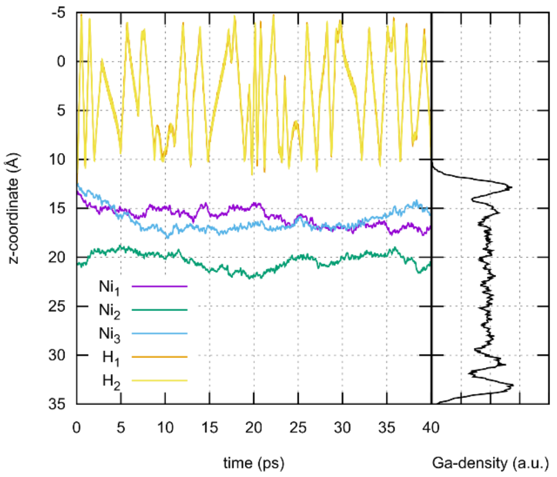

(b) trajectory 2

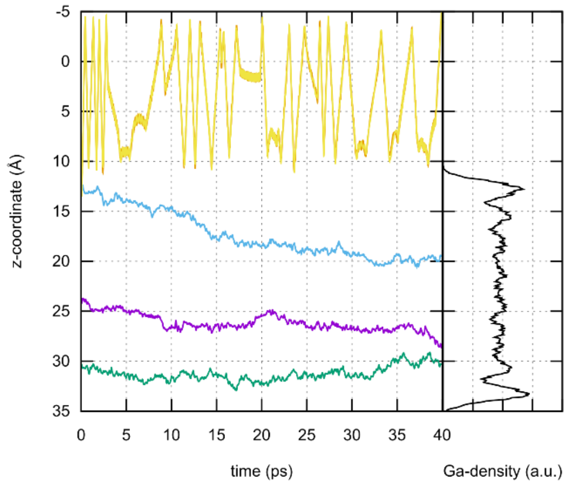

(c) trajectory 3

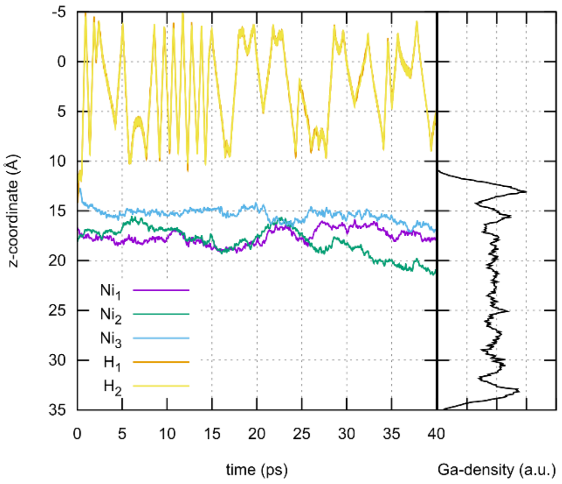

(d) trajectory 4

Figure S25: Time dependent z-coordinates of all Ni and H atoms obtained from AIMD simulations of Ga-Ni surface slabs with one Ni (Ni_3) initially placed at the surface with a H_2 molecule on it and two other Ni atoms (Ni_1 and Ni_2) placed in the bulk region.

References

[1] A. Søgaard, A. L. De Oliveira, N. Taccardi, M. Haumann, P. Wasserscheid, *Catal Sci Technol* **2021**, *11*, 7535–7539.

[2] A. Søgaard, Ga-Ni Supported Catalytically Active Liquid Metal Solutions (SCALMS) for Selective Alkene Oligomerisation, Doctoral thesis, Friedrich-Alexander-Universität Erlangen-Nürnberg (FAU), Technische Fakultät, **2024**.

[3] N. Raman, M. Wolf, M. Heller, N. Heene-Würl, N. Taccardi, M. Haumann, P. Felfer, P. Wasserscheid, *ACS Catal* **2021**, *11*, 13423–13433.

[4] O. Sebastian, A. Al-Shaibani, N. Taccardi, U. Sultan, A. Inayat, N. Vogel, M. Haumann, P. Wasserscheid, *Catal Sci Technol* **2023**, *13*, 4435.

[5] K. Lips, D. E. Starr, M. Bar, T. F. Schulze, F. Fenske, S. Christiansen, R. van de Krol, S. Raoux, G. Reichardt, F. Schafers, S. Hendel, R. Follath, J. Bahrdt, M. Scheer, G. Wustefeld, P. Kuske, M. Havecker, A. Knop-Gericke, R. Schlogl, B. Rech, in *2014 IEEE 40th Photovoltaic Specialist Conference (PVSC)*, IEEE, **2014**, pp. 698–700.

[6] M. B. Trzhaskovskaya, V. K. Nikulin, V. I. Nefedov, V. G. Yarzhemsky, *Opt Spectrosc* **2004**, *96*, 765–773.

[7] M. B. Trzhaskovskaya, V. I. Nefedov, V. G. Yarzhemsky, *At Data Nucl Data Tables* **2001**, *77*, 97–159.

[8] H. Shinotsuka, S. Tanuma, C. J. Powell, D. R. Penn, *Surface and Interface Analysis* **2015**, *47*, 871–888.

[9] H. Shinotsuka, B. Da, S. Tanuma, H. Yoshikawa, C. J. Powell, D. R. Penn, *Surface and Interface Analysis* **2017**, *49*, 238–252.

[10] H. Shinotsuka, S. Tanuma, C. J. Powell, D. R. Penn, *Surface and Interface Analysis* **2019**, *51*, 427–457.

[11] W. S. M. Werner, W. Smekal, C. J. Powell, *Simulation of Electron Spectra for Surface Analysis (SESSA) Version 2.2.0 User s Guide*, **2021**.

[12] G. Kresse, D. Joubert, *Phys Rev B* **1999**, *59*, 1758–1775.

[13] G. Kresse, J. Furthmüller, *Comput Mater Sci* **1996**, *6*, 15–50.

[14] G. Kresse, J. Furthmüller, *Phys Rev B* **1996**, *54*, 11169–11186.

[15] J. P. Perdew, K. Burke, M. Ernzerhof, *Phys Rev Lett* **1996**, *77*, 3865–3868.

[16] M. Methfessel, A. T. Paxton, *Phys Rev B* **1989**, *40*, 3616–3621.

[17] W. Tang, E. Sanville, G. Henkelman, *Journal of Physics: Condensed Matter* **2009**, *21*, 084204.

[18] G. Henkelman, A. Arnaldsson, H. Jónsson, *Comput Mater Sci* **2006**, *36*, 354–360.

[19] P. E. Blöchl, O. Jepsen, O. K. Andersen, *Phys Rev B* **1994**, *49*, 16223–16233.

[20] R. Jinnouchi, J. Lahnsteiner, F. Karsai, G. Kresse, M. Bokdam, *Phys Rev Lett* **2019**, *122*, 225701.

[21] R. Jinnouchi, F. Karsai, G. Kresse, *Phys Rev B* **2019**, *100*, 014105.

[22] R. Jinnouchi, F. Karsai, C. Verdi, R. Asahi, G. Kresse, *J Chem Phys* **2020**, *152*, 234102.

[23] M. Grabau, S. Krick Calderón, F. Rietzler, I. Niedermaier, N. Taccardi, P. Wasserscheid, F. Maier, H.-P. Steinrück, C. Papp, *Surf Sci* **2016**, *651*, 16–21.

[24] H. Wittkämper, S. Maisel, M. Wu, J. Frisch, R. G. Wilks, M. Grabau, E. Spiecker, M. Bär, A. Görling, H.-P. Steinrück, C. Papp, *J Chem Phys* **2020**, *153*, DOI 10.1063/5.0021647.

[25] L. P. H. Jeurgens, W. G. Sloof, F. D. Tichelaar, E. J. Mittemeijer, *Surf Sci* **2002**, *506*, 313–332.
